# Harnessing PET to track micro- and nanoplastics in vivo

**DOI:** 10.1038/s41598-021-90929-6

**Published:** 2021-06-01

**Authors:** Outi Keinänen, Eric J. Dayts, Cindy Rodriguez, Samantha M. Sarrett, James M. Brennan, Mirkka Sarparanta, Brian M. Zeglis

**Affiliations:** 1 Department of Chemistry, Hunter College, City University of New York, 413 East 69th Street, New York, NY 10021 USA; 2 Department of Chemistry, University of Helsinki, Helsinki, Finland; 3 Department of Radiology, Memorial Sloan Kettering Cancer Center, New York, NY USA; 4 Ph.D. Program in Chemistry, Graduate Center of the City University of New York, New York, NY USA; 5 Ph.D. Program in Biochemistry, Graduate Center of the City University of New York, New York, NY USA; 6 Department of Radiology, Weill Cornell Medical College, New York, NY USA

**Keywords:** Nanotoxicology, Environmental sciences, Nuclear chemistry

## Abstract

The proliferation of plastics in the environment continues at an alarming rate. Plastic particles have been found to be persistent and ubiquitous pollutants in a variety of environments, including sea water, fresh water, soil, and air. In light of this phenomenon, the scientific and medical communities have become increasingly wary of the dangers posed to human health by chronic exposure to microplastics (< 5 mm diameter) and nanoplastics (< 100 nm diameter). A critical component of the study of the health effects of these pollutants is the accurate determination of their pharmacokinetic behavior in vivo. Herein, we report the first use of molecular imaging to track polystyrene (PS) micro- and nanoplastic particles in mammals. To this end, we have modified PS particles of several sizes—diameters of 20 nm, 220 nm, 1 µm, and 6 µm—with the chelator desferrioxamine (DFO) and radiolabeled these DFO-bearing particles with the positron-emitting radiometal zirconium-89 (^89^Zr; t_1/2_ ~ 3.3 d). Subsequently, positron emission tomography (PET) was used to visualize the biodistribution of these radioplastics in C57BL/6J mice at 6, 12, 24, and 48 h after ingestion. The imaging data reveal that the majority of the radioplastics remain in the gastrointestinal tract and are eliminated through the feces by 48 h post-ingestion, a result reinforced by acute biodistribution studies. Ultimately, this work suggests that nuclear imaging—and PET in particular—can be a sensitive and effective tool in the urgent and rapidly growing effort to study the in vivo behavior and potential toxicity of micro- and nanoplastics.

## Introduction

Over the last decade, micro- and nanoplastic pollution has become recognized as a global environmental threat and a possible health hazard to humans^1–4^. Micro- and nanoplastics have been found in a diverse array of environments, including oceans, rivers, sediments, sewage, soil, and even the air^1,5–10^. Even more concerningly, they have been isolated from tap water, bottled water, table salt, and seafood^11–15^. Recent studies have even demonstrated their human consumption by uncovering the presence of microplastics in human fecal matter^16^. Even more recently and concerningly microplastics were isolated from human placenta^17^. In order to understand the pathophysiological consequences of micro- and nanoplastic pollution, numerous investigations into the bioaccumulation of plastics in aquatic animals, invertebrates, and seabirds have been conducted^5^. Recent work in aquatic organisms has shown that micro- and nanoplastics can alter gene expression, induce oxidative stress, elicit immune responses, cause genotoxicity, disrupt the endocrine system, trigger metabolic disorders, and lead to neurotoxicity, reproductive abnormities, and other trans-generational effects^18–28^.

Only a handful of investigations have focused on the fate of plastic particles in mammals^29,30^. In 2017, Deng et al*.* were the first to study the toxicity and bioaccumulation of polystyrene (PS) microplastics in mice^31^. They observed the accumulation of fluorescent PS in the liver, kidneys, and gut and determined that exposure to microplastics disrupted lipid metabolism, induced oxidative stress, and produced signs of neurotoxicity. This investigation was later contradicted by the work of Stock et al*.* (2019) and Rafiee et al. (2018) in which translocation of PS particles from the gut was not observed and toxicologically relevant effects related to PS exposure were not found in vivo in rodents or in vitro in human intestinal epithelium^32,33^. Even more recently, however, several studies have suggested that microplastics can induce gut microbiota dysbiosis, intestinal barrier dysfunction, inflammation, and metabolic disorders in both mice and their offspring^34–46^.

**Figure 1:**
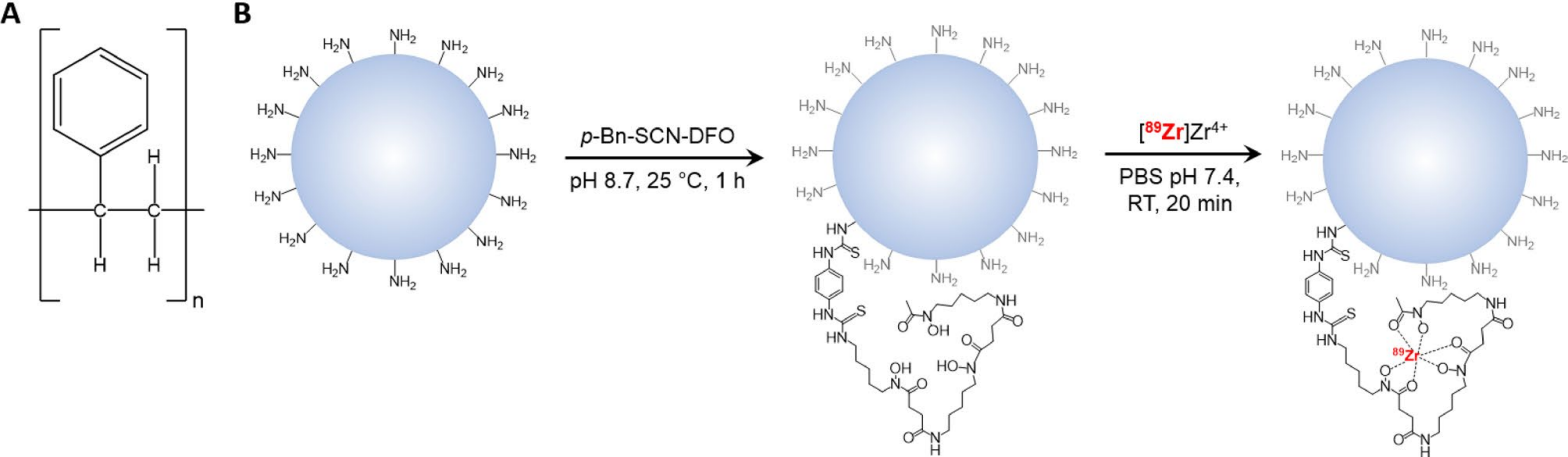
(**A**) The structure of polystyrene (PS). (**B**) The modification of an amine-functionalized PS particle with desferrioxamine (DFO) and its subsequent radiolabeling with [^89^Zr]Zr^4+^ to yield [^89^Zr]Zr-DFO-PS.

Herein we report the first use of positron emission tomography (PET) to track the biological behavior of micro- and nanoplastics in vivo. The over-arching goal of this investigation is the development of a new method for the non-invasive, quantitative study of the pharmacokinetics of micro- and nanoplastics that could be used to shed light on the controversies enumerated above. As we have noted, previous attempts at elucidating the in vivo behavior of microplastics have relied upon fluorophore-labeled particles. Yet this approach has several critical drawbacks, including the poor tissue penetration of fluorescence, the lack of a quantifiable output, the perturbation of the surface of the particle with bulky fluorophores, and the inherent instability of the fluorophore-particle linkage in vivo^47–51^. In contrast, our novel strategy is predicated on modifying micro- and nanoplastic particles with a chelator (desferrioxamine, DFO) and subsequently radiolabeling them with the long-lived positron-emitting radiometal zirconium-89 (^89^Zr; t_1/2_ ~ 3.3 d) (Fig. 1). This approach produces radiolabeled plastic particles (radioplastics) that are stable both in vitro and in vivo and are only minimally altered compared to their parent particle. Even more importantly, these radioplastics can be harnessed for non-invasive, longitudinal, and quantitative PET imaging experiments. To date, only a single other study has employed radioactivity for the study of microplastics: carbon-14 (^14^C, t_1/2_ ~ 5,730 y) was used to study the ex vivo biodistribution of PS microplastics in scallops^52^. Ultimately, we are confident that PET can play a significant role in the exploration of the pharmacokinetic profiles of micro- and nanoplastics as well as the pathophysiological effects of acute and chronic exposure to these pollutants. It is our hope that this investigation spurs interest in the use of nuclear imaging as a tool to study not only plastics but other environmental pollutants as well.

## Methods

Unless otherwise noted, all reagents were purchased from commercial suppliers and used without further purification. All water used was ultrapure water (18.2 MΩcm). To make Chelex-treated ultrapure water (Chelex-H_2_O) or Chelex-treated phosphate buffered saline pH 7.4 (Chelex-PBS), 5 g of Chelex 100 Resin (100–200 mesh, sodium form, Bio-Rad) was added to 1 L of solution, and the mixture was then stirred overnight. Subsequently, the resin was allowed to settle and removed via filtration. All experiments involving laboratory animals were performed in accordance with a protocol approved by the Institutional Animal Care and Use Committees of Hunter College, Weill Cornell Medical College, and Memorial Sloan Kettering Cancer Center. All experiments were performed in accordance with relevant guidelines and regulations and complied with ARRIVE.

### Modification of PS particles with DFO

Amine-functionalized polystyrene particles (NH_2_-PS) with diameters of 220 nm, 1 µm, and 6 µm were purchased from Spherotech, Inc. (Lake Forest, IL, USA), while amine-functionalized polystyrene particles with a diameter of 20 nm were purchased from Nanocs, Inc. (New York, NY, USA). To begin, the 220 nm, 1 µm, and 6 µm NH_2_-PS particles (5 mg each) were centrifuged (10,000*g*, 5 min), and the supernatant was discarded. The particles were then resuspended in 1 mL Chelex-H_2_O, the particles were centrifuged a second time (10,000*g*, 5 min), and the supernatant was again discarded. This wash with Chelex-H_2_O was repeated a second time. After two Chelex-H_2_O washes, the particles were suspended in 0.5 mL Chelex-H_2_O (0.5 ml) with 25% DMSO. In contrast, 5 mg of the 20 nm NH_2_-PS particles were washed using Chelex-PBS with 0.05% tween-20 (Chelex-PBS-T; used to prevent aggregation and adsorption on the filter) and Amicon Ultra-15 centrifugal filters with a 100 kDa cut-off. More specifically, the 20 nm particles were placed in the filter unit, and the filter was filled with Chelex-PBS-T prior to centrifuging at 3500*g* for 20 min. The residual solution (~ 0.3 mL) was then recovered from the filter unit, and 0.1 mL of DMSO was added.

In each case, the pH of the solution of particles—5 mg NH_2_-PS in 0.5 mL of Chelex-PBS with 25% DMSO—was adjusted to pH 8.7 with 0.1 M Na_2_CO_3_, and 0.5 mg of *p*-SCN-Bn-DFO (20 µL; 25 mg/mL in DMSO; Macrocyclics, Inc.) was then added. The vials were put on a thermomixer at 25 °C and 750 rpm for 1 h. Subsequently, the 220 nm, 1 µm, and 6 µm particles were washed five times with 1 mL of 25% DMSO in Chelex-H_2_O as described above and finally suspended in Chelex-H_2_O to bring the final concentration to 5 mg/mL. The reaction mixture of 20 nm particles, in contrast, was placed in an Amicon filter containing Chelex-PBS-T with 25% DMSO before centrifuging at 3500*g* for 5 min. After centrifugation, the flow-through was discarded, the filter was re-filled with Chelex-PBS-T with 25% DMSO, and the unit was centrifuged at 3500*g* for 5 min. This centrifugation step was then repeated a third time before the particle solution was collected from the unit, and Chelex-PBS-T was added to bring the final concentration to 5 mg/mL.

### Radiolabeling of DFO-PS

^89^Zr-oxalate in 1.0 M oxalic acid was adjusted to pH 7.0 with 1.0 M Na_2_CO_3_. After the evolution of CO_2_ ceased, an aliquot of this ^89^Zr solution (92.5 MBq, 2.5 mCi, 0.15 mL) was mixed with each DFO-PS suspension [2.5 mg in 0.5 mL of Chelex-H_2_O (220 nm, 1 µm, and 6 µm particles) or Chelex-PBS-T (20 nm particles)]. The reaction mixtures were then incubated on a thermomixer at 37 °C and 700 rpm for 20 min. After incubation, the crude radiolabeling yield was determined with iTLC using a glass microfiber chromatography paper impregnated with silica gel (Agilent, Inc.) and an eluent of 50 mM ethylenediaminetetraacetic acid (EDTA, pH 5.5). EDTA rapidly chelates any free zirconium, and the resultant [^89^Zr]Zr-EDTA travels with the solvent front (*i.e.* Rf_[89Zr]Zr-EDTA_ = 0.9–1). In contrast, ^89^Zr bound to the plastic particles remains at the origin (*i.e.* Rf_[89Zr]Zr-DFO-PS_ = 0). To make sure no free ^89^Zr was adsorbed to the surface of the DFO-modified PS, the particles were washed before they were administered to animals. To this end, the 220 nm, 1 µm, and 6 µm particles were washed two times with 1 mL of 50 mM EDTA pH 5.5 solution and once with 1 mL of PBS before suspending them in PBS. In the case of the 20 nm particles, the reaction mixture was placed in an Amicon filter unit (100,000 Da MWCO) filled with 2 mL of 50 mM EDTA pH 5.5, and the unit was centrifuged at 3500*g* for 5 min. The residual was placed in an Amicon filter unit (100,000 Da MWCO) filled with PBS-T, and the unit was centrifuged at 3500*g* for 20 min. After centrifugation, the flow-through was discarded, and the particles were suspended in PBS-T. The final concentration for each [^89^Zr]Zr-DFO-PS suspension was adjusted to 1 mg/mL and 18.5 MBq/mL (0.5 mCi/mL).

### In vitro stability of [^89^Zr]Zr-DFO-PS

The in vitro stability of [^89^Zr]Zr-DFO-PS was studied in PBS pH 7.4, simulated gastric fluid pH 3.0 (SGF; Ricca Chemical Company, Arlington, TX, USA), and simulated intestinal fluid pH 6.0 (SIF; Ricca Chemical Company, Arlington, TX, USA) in triplicate. The radiolabeled PS particles (0.05 mL) were mixed with 1 mL of PBS, SGF, or SIF. At predetermined timepoints, iTLC was performed to determine the amount of free [^89^Zr]Zr^4+^ using a glass microfiber chromatography paper impregnated with silica gel (Agilent, Inc.) and an eluent of 50 mM EDTA pH 5.5.

### Characterization of the DFO-modified PS particles

After each modification of the NH_2_-PS particles, the size and zeta potential of the DFO-bearing particles were determined using a Malvern Zetasizer Nano ZS. To compare the characteristics of plain PS to those of NH_2_-modified particles, plain PS with sizes of 20 nm, 220 nm, 1 µm, and 5 µm were purchased from the same vendors (Nanocs, Inc.; New York, NY, USA; and Spherotech, Inc.; Lake Forest, IL, USA). The size of the 220 nm, 1 µm, and 6 µm particles was measured in water, while that of the 20 nm particles was measured in PBS-T. PBS-T was used with the 20 nm particles because the particles aggregated and adsorbed onto the Amicon filter unit during purification if only water were used. In every case, the zeta potential was measured in 10 mM phosphate buffer pH 7.0. The following parameters were used in the analysis: absorption of PS = 0.01, refractive index (RI) of PS = 1.59, RI of water = 1.33, RI of PBS-T = 1.34, RI of 10 mM phosphate buffer pH 7 = 1.333, viscosity of water = 0.8872 cP, viscosity of PBS-T = 1 cP, viscosity of 10 mM phosphate buffer pH 7 = 1 cP, and dielectric constant of 10 mM phosphate buffer pH 7 = 78.4.

### In vivo studies

8–9-week-old C57BL/6 J female mice were purchased from The Jackson Laboratory and allowed to acclimatize for approximately 1 week prior to experimentation. Animals were group housed in ventilated cages (#9, Thoren Caging Systems, Hazleton, Pennsylvania, USA) with water (reverse osmosis-filtered and acidified) and food (PicoLab Rodent Diet 20, LabDiet, St. Louis, MO, USA) ad libitum. Lighting was set to a 12:12 rhythm, and the temperature was maintained at 22 ± 1 °C.

#### Ex vivo* biodistribution*

The mice were fasted for 18 h before the administration of [^89^Zr]Zr-DFO-PS (1.85 MBq, 50 µCi, 18.5 MBq/mg, 0.1 mg in 0.1 mL of PBS or PBS-T), [^89^Zr]Zr-DFO (1.85 MBq, 50 µCi, 14.0 MBq/µmol, 0.1 mg in 0.1 mL of PBS), or [^89^Zr]Zr^4+^ (1.85 MBq, 50 µCi, in 0.1 mL of PBS) via a stainless-steel feeding needle *per os*. Water was available to the mice during fasting, and food was made available immediately after the administration of the radiolabeled material. At 6, 12, 24, and 48 h post injection, mice (n = 5) were euthanized via asphyxiation using CO_2_(g), and selected tissues were collected and placed into pre-weighed tubes. The mass of each organ was determined, and each sample was then counted using a Wizard^2^ automatic gamma counter. Four aliquots (5 µL) were weighed and counted as internal standards for each radiolabeled construct. The total injected dose was found as the mass injected dose × internal standard average counts/g. The percent injected dose (%ID) was determined as the counts for the tissue × 100/total injected dose. The %ID/g was calculated as the %ID/tissue mass in g.

#### PET imaging

PET imaging was carried out on an Inveon PET/CT scanner (Siemens) or a microPET Focus 120 scanner (Concorde Microsystems). The mice were fasted for 18 h before the administration of [^89^Zr]Zr-DFO-PS (1.85 MBq, 50 µCi, 18.5 MBq/mg, 0.1 mg in 0.1 mL of PBS or PBS-T), [^89^Zr]Zr-DFO (1.85 MBq, 50 µCi, 14.0 MBq/µmol, 0.1 mg in 0.1 mL of PBS), and [^89^Zr]Zr^4+^ (1.85 MBq, 50 µCi, in 0.1 mL of PBS) via a stainless-steel feeding needle *per os*. Water was available during fasting, and food was made available immediately after the administration of the radiotracer. Mice were anaesthetized with 2% isoflurane/medical air inhalation approximately 5 min prior to recording the PET images and kept under anesthesia during the PET scan. Data was acquired as static images at 6, 12, 24, and 48 h after the administration. The energy and coincidence timing windows were 350–750 keV and 6 ns, respectively. Data were sorted into two-dimensional histograms by Fourier re-binning, and transverse images were reconstructed by filtered back-projection (FBP). The image data were normalized to correct for non-uniformity of response of the detector, dead-time count losses, positron branching ratio, and physical decay to the time of injection, but no attenuation, scatter, or partial volume averaging correction was applied. Images were analyzed by using ASIPro VM software (Concorde Microsystems).

#### In vivo* stability*

To determine the in vivo stability of each [^89^Zr]Zr-DFO-PS, the contents of stomach, small intestines, and large intestines were separated from the organs and used to perform two experiments. In each case, the contents of the gastrointestinal tract were mixed with PBS (1 mL) and homogenized. EDTA (0.2 mL, 50 mM, pH 5.5) was then added, and iTLC was run. Second, the contents of all three sections were combined and placed in an Amicon Ultra-15 filter unit with a 100 kDa cut-off, and the filter unit was filled with PBS. The unit was then centrifuged at 3500*g* for 30 min, and the flow through was collected. This procedure was repeated two more times, and the flow-through was collected each time. The radioactivity in the combined flow-through as well as that remaining in the filter unit were measured with a dose calibrator or Wizard^2^ automatic gamma counter calibrated with ^89^Zr. Any radioactivity in the combined flow-through—which must, by definition, be part of a low molecule weight species—was considered to be the result of in vivo decomposition.

## Results

### Conjugation, radiolabeling, and characterization

The first step in this investigation was the covalent attachment of the radiometal chelator desferrioxamine (DFO) to the primary amines decorating the surface of the NH_2_-PS particles. Subsequently, the radiolabeling of DFO-bearing micro- and nanoplastics was carried out by mixing [^89^Zr]Zr^4+^ (92.5 MBq, 2.5 mCi, 0.15 mL) with a suspension of each DFO-PS [2.5 mg in 0.5 mL of Chelex-H_2_O (220 nm, 1 µm, and 6 µm particles) or Chelex-PBS-T (20 nm particles)] and incubating the reaction mixture at 37 °C and 700 rpm for 20 min. In each case, the crude radiolabeling yield was determined via iTLC to be > 99%. However, to make sure no free [^89^Zr]Zr^4+^ was adsorbed to the surface of the particles, each [^89^Zr]Zr-DFO-PS was washed before they were administered to animals (see “Methods”). The isolated radiochemical yield for each ^89^Zr-labeled radioplastic was 92%, 96%, 98%, and 95% for the 20 nm, 220 nm, 1 µm, and 6 µm variants, respectively. The final concentration of each [^89^Zr]Zr-DFO-PS suspension was adjusted to be 1 mg/mL and 18.5 MBq/mL (0.5 mCi/mL).

We next performed size, polydispersity index (PdI), and zeta potential measurements in order to confirm that the fundamental physiochemical properties of the NH_2_-PS particles were not altered by the modification of their surfaces with chelators and radionuclides. Table 1 summarizes this size, PdI, and zeta potential data. Critically, the PdI, zeta potential, and particle size remained largely the same within each set of NH_2_-PS, PS-DFO, and [^89^Zr]Zr-DFO-PS particles despite the attachment of the chelator and radiometal. Completely unmodified PS particles with 20 nm, 220 nm, 1, and 5 µm diameters were used to probe for differences between the bare and functionalized plastics. In this case, the zeta potentials of each of the NH_2_-PS particles proved higher than that of their completely unmodified cousins: − 40 to − 50 mV for the former compared to − 85 mV for the latter. This difference is not entirely surprising given the charge that the primary amines confer upon the surface of the particle. These small differences aside, the relatively high negative surface charge across all of the particle constructs reflects stable formulations without large aggregates.

**Table 1:** Size, polydispersity index (PdI), and zeta potential measurements of the unmodified PS, NH_2_-PS, DFO-PS, and [^89^Zr]Zr-DFO-PS particles.

Particle^a^	Size (nm)	PdI	Zeta potential (mV)
PS (20 nm)	30.6 ± 0.3	0.12 ± 0.01	− 83.7 ± 3.0
NH_2_-PS (20 nm)	24.8 ± 0.2	0.14 ± 0.02	− 37.3 ± 5.6
DFO-PS (20 nm)	25.7 ± 0.2	0.18 ± 0.01	− 38.8 ± 1.7
[^89^Zr]Zr-DFO-PS (20 nm)	24.6 ± 0.1	0.12 ± 0.01	− 40.4 ± 5.5
PS (220 nm)	251 ± 5	0.46 ± 0.02	− 73.7 ± 2.4
NH_2_-PS (220 nm)	206 ± 10	0.46 ± 0.02	− 49.7 ± 2.6
DFO-PS (220 nm)	201 ± 12	0.43 ± 0.07	− 41.5 ± 1.3
[^89^Zr]Zr-DFO-PS (220 nm)	230 ± 15	0.46 ± 0.09	− 48.3 ± 1.8
PS (1.04 µm)	1023 ± 11	0.12 ± 0.05	− 86.5 ± 4.6
NH_2_-PS (1.13 µm)	1116 ± 4	0.14 ± 0.06	− 51.2 ± 2.1
DFO-PS (1.13 µm)	1136 ± 28	0.07 ± 0.03	− 56.5 ± 4.3
[^89^Zr]Zr-DFO-PS (1.13 µm)	1064 ± 34	0.28 ± 0.01	− 58.5 ± 2.9
PS (5.09 µm)	4814 ± 158	0.07 ± 0.03	− 84.8 ± 5.1
NH_2_-PS (6.22 µm)	6118 ± 98	0.10 ± 0.08	− 42.1 ± 1.5
DFO-PS (6.22 µm)	6103 ± 326	0.28 ± 0.09	− 43.6 ± 6.2
[^89^Zr]Zr-DFO-PS (6.22 µm)	6305 ± 168	0.32 ± 0.08	− 41.3 ± 0.7

^a^The size in parentheses is the size reported by the manufacturer.

### In vitro stability of [^89^Zr]Zr-DFO-PS particles

**Figure 2:**
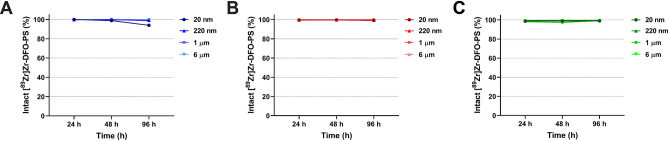
In vitro stability of [^89^Zr]Zr-DFO-PS particles in (**A**) phosphate buffered saline (PBS, pH 7.4), (**B**) simulated gastric fluid (SGF, pH 3.0), and (**C**) simulated intestinal fluid (SIF, pH 6.0). All experiments were performed in triplicate. The values are presented as mean ± standard deviation.

The stability of the [^89^Zr]Zr-DFO-PS particles was first interrogated in phosphate buffered saline pH 7.4 (PBS), with each of the ^89^Zr-labeled micro- and nanoplastics remaining > 95% intact over the course of 4 d at 37 °C. Assays were also performed in simulated gastric fluid (SGF; pH 3) and simulated intestinal fluid (SIF; pH 6) to investigate the stability of the [^89^Zr]Zr-DFO-PS particles in the gastrointestinal tract. Even under these lower pH conditions, the radioplastics remained remarkably stable: each proved to be > 99% intact after 4 days at 37 °C (Fig. 2 and Table S1).

### Biodistribution of radioplastics in mice

**Figure 3:**
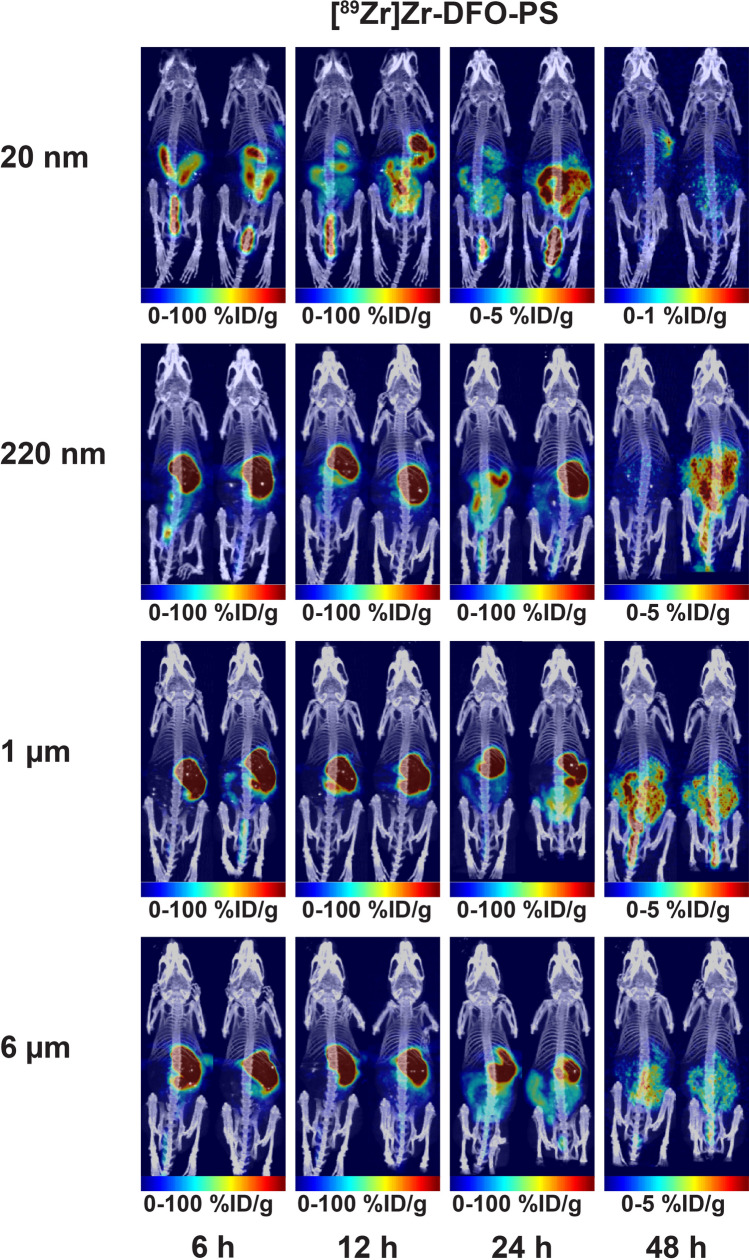
Maximum intensity projection (MIP) PET-CT images of mice that received 1.85 MBq (50 µCi) of [^89^Zr]Zr-DFO-PS *per os*. Two mice are shown for each particle size (n = 4 in each cohort). See *Fig. S1* for transverse, coronal, and sagittal slices of the sections with the highest activity concentrations.

**Figure 4:**
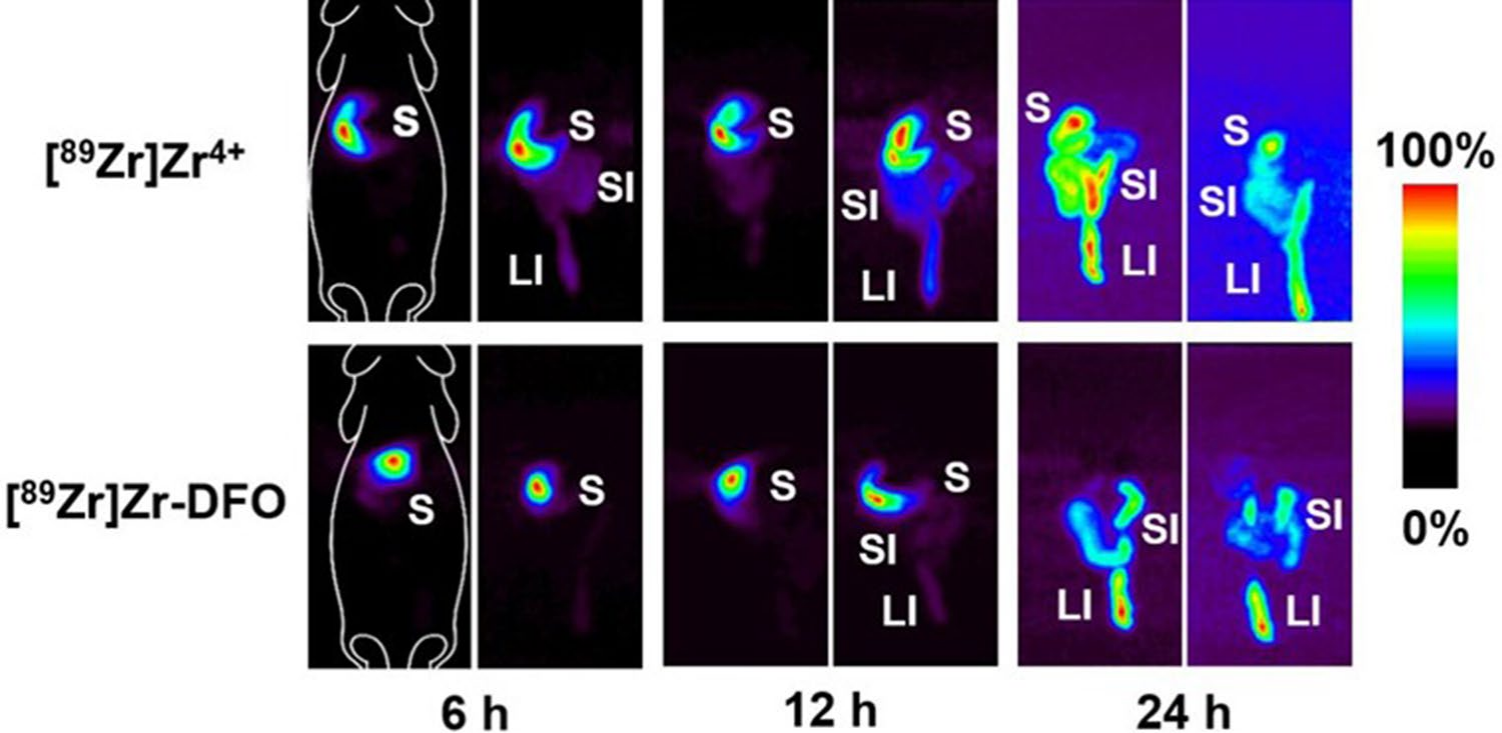
MIP PET images of control mice that received 1.85 MBq (50 µCi) of [^89^Zr]Zr^4+^ or [^89^Zr]Zr-DFO *per os.* Two mice from each cohort are shown (n = 4). S = stomach. SI = small intestine. LI = large intestine.

**Figure 5:**
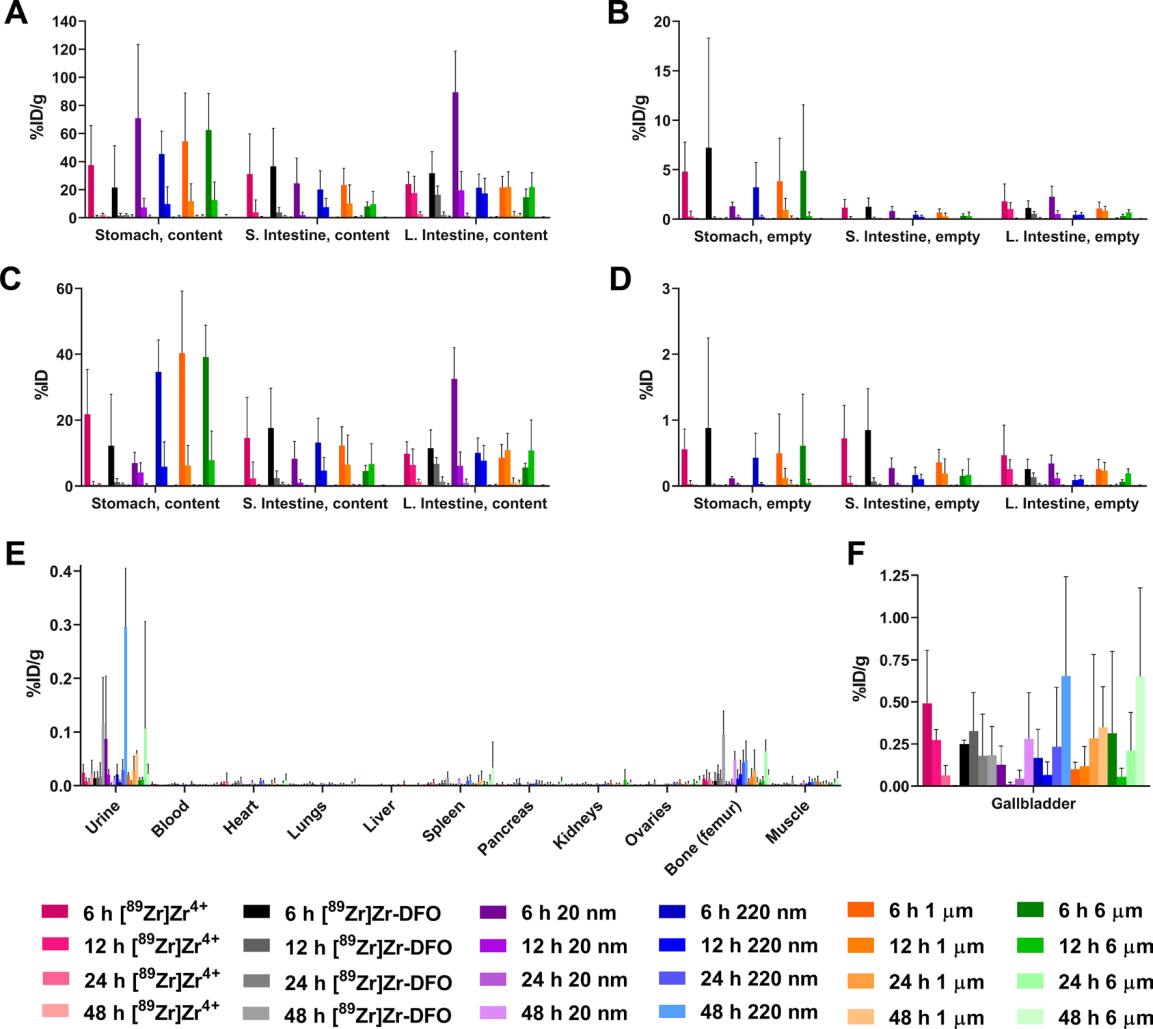
Ex vivo biodistribution data acquired after the oral administration of [^89^Zr]Zr^4+^, [^89^Zr]Zr-DFO, and four sizes of [^89^Zr]Zr-DFO-PS particles (20 nm, 220 nm, 1 μm, and 6 μm). (**A,B**) %ID/g data for the gastrointestinal tract; (**C,D**) %ID data for the gastrointestinal tract; (**E**) %ID/g data for organs other than gastrointestinal tract and gallbladder; (**F**) %ID/g data for the gallbladder. The data are presented as mean ± standard deviation. S. Intestine = Small Intestine. L. Intestine = Large Intestine. Numerical biodistribution data can be found in Tables S2-S13*.*

With the radioplastics synthesized and characterized, the next step was to explore their behavior in healthy, immunocompetent mice via PET imaging and biodistribution experiments. To this end, the mice were administered 1.85 MBq [^89^Zr]Zr-DFO-PS (0.1 mg), [^89^Zr]Zr-DFO (0.1 mg), or [^89^Zr]Zr^4+^ orally via a gavage needle. In order to reduce inter-individual variation due to differing gastric emptying rates, the mice were fasted for 18 h prior to the administration of the ^89^Zr-labeled compounds. PET images collected at 6, 12, 24, and 48 h clearly visualized the movement of [^89^Zr]Zr-DFO-PS, [^89^Zr]Zr-DFO and ^89^Zr through the gastrointestinal tract, with the highest activity concentrations in the large intestines by the later time points (Figs. 3, 4, and S1). The ex vivo biodistribution data confirmed the observations from the PET images: in each case, the overwhelming majority of the radioactivity remained in the contents of gastrointestinal tract and was subsequently eliminated through the feces (Fig. 5 and Tables S2–S13). Perhaps not surprisingly, the control compounds—[^89^Zr]Zr-DFO and [^89^Zr]^4+^—and the smallest [^89^Zr]Zr-DFO-PS particles (20 nm) travelled through the gastrointestinal tract faster than the 220 nm, 1 µm, and 6 µm [^89^Zr]Zr-DFO-PS particles.

The gall bladder was the only organ in which there was a dramatic difference in uptake between the controls and the [^89^Zr]Zr-DFO-PS particles. To wit, free [^89^Zr]Zr^4+^ exhibited a moderate level of uptake in the gallbladder at 6 h (0.5 ± 0.3%ID/g) that gradually decreased to background levels by 48 h, while the uptake of [^89^Zr]Zr-DFO in the gallbladder remained largely static throughout the experiment. In contrast, the uptake of each of the [^89^Zr]Zr-DFO-PS particles in the gallbladder reached a maximum at 48 h after administration. It is important to note, however, that the perceived differences in the activity concentrations of free [^89^Zr]Zr^4+^, [^89^Zr]Zr-DFO, and [^89^Zr]Zr-DFO-PS in the gallbladder do not hold up under more intense scrutiny, as there is no statistically significant difference at any time point between the cohorts (using a two-tailed t-test with Welch’s correction carried out with GraphPad Prism 9.0). Ultimately, we hypothesize that the variability observed in the gallbladder uptake of the various ^89^Zr-labeled constructs may be the result of inter-individual differences stemming from whether each animal’s gallbladder was full or empty at the time of euthanasia.

### In vivo stability of [89Zr]Zr-DFO-PS particles

**Figure 6:**
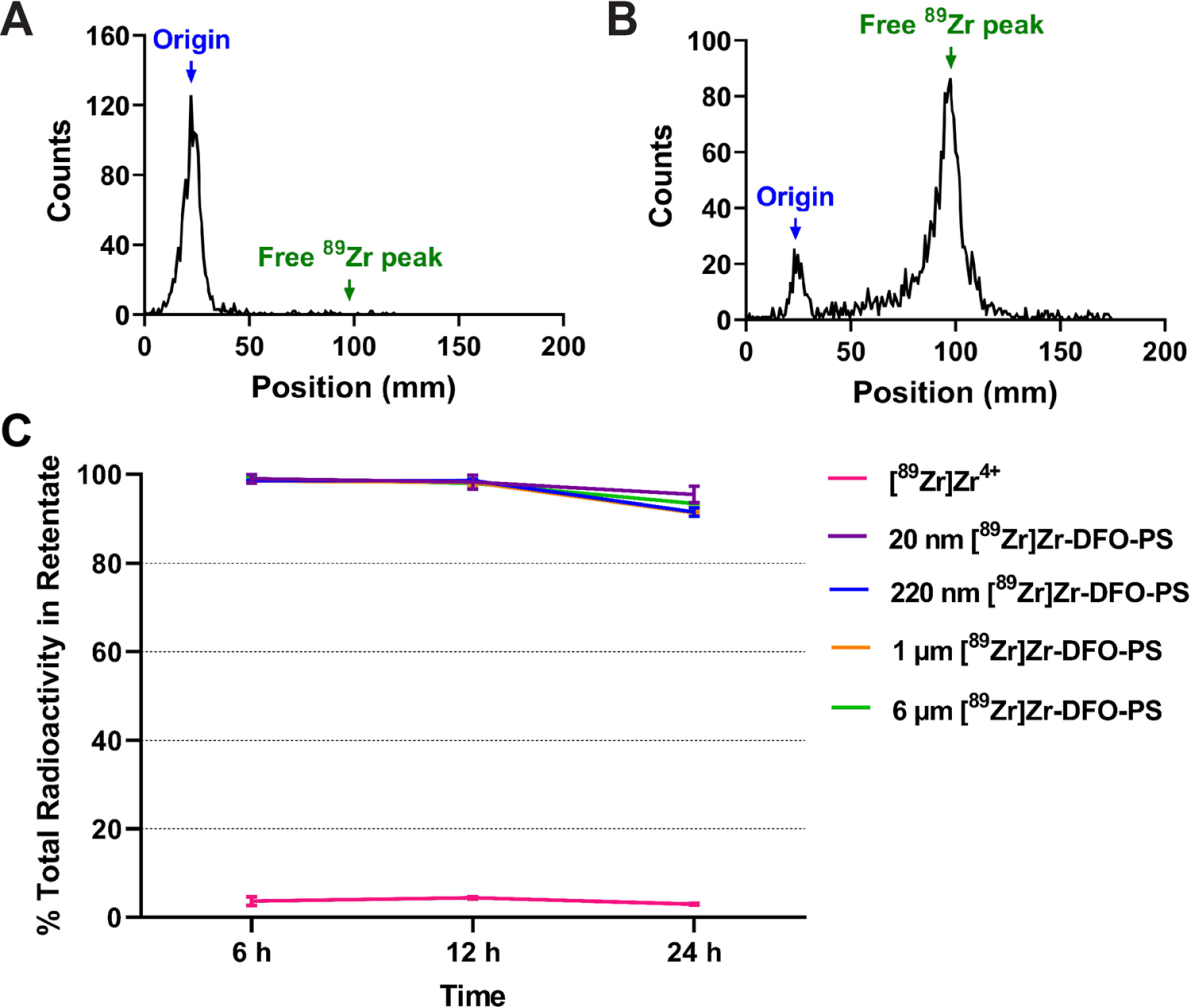
The in vivo stability of [^89^Zr]Zr-DFO-PS particles. Representative iTLC chromatograms of the homogenized gastrointestinal contents of mice collected 12 h after the administration *per os* of **(A)** [^89^Zr]Zr-DFO-PS and **(B)** [^89^Zr]Zr^4+^. (**C**) The percentage of the total radioactivity remaining in the retentate after the centrifugal filtration (Amicon Ultra-15; 100 kDa molecular weight cut-off) of the contents of the gastrointestinal tracts of mice collected 6, 12, and 24 h after the administration *per os* of [^89^Zr]Zr-DFO-PS (20 nm, 220 nm, 1 µm or 6 µm) or [^89^Zr]Zr^4+^.

The in vivo stability of [^89^Zr]Zr-DFO-PS was studied using two different analytical methods: iTLC and size exclusion filtration. In the iTLC assay, the contents of the stomach, small intestines and large intestines were collected at different points after administration and subsequently homogenized with PBS. Subsequent iTLCs—run using glass microfiber chromatography paper impregnated with silica gel and an eluent of 50 mM EDTA pH 5.5—of each ^89^Zr-labeled radioplastic performed 6, 12, and 24 h after administration revealed that > 99% of the radioactivity remained at the origin and thus no free ^89^Zr could be detected in the sample (Fig. 6A). It should also be noted that iTLCs performed on samples from animals that had received free [^89^Zr]Zr^4+^ exhibit 5–15% of the total radioactivity at the origin, suggesting that some of the radiometal may become associated with proteins and other biomolecules (Fig. 6B). In the size exclusion assay, the contents of the entire gastrointestinal tract were collected and placed in an Amicon Ultra-15 filtration unit with a 100 kDa cut-off. The filter unit was then centrifuged twice, each time adding PBS and collecting the flow-through. Any activity in the flow-through—which must, by definition, have a molecular weight < 100,000 Da—was assumed to originate from free [^89^Zr]Zr^4+^ or ^89^Zr-labeled, low molecular weight decomposition products. In each of the [^89^Zr]Zr-DFO-PS samples, > 90% of the total radioactivity remained in the retentate after the entire observation period (24 h). In contrast, only ~ 5% of the total radioactivity remained in the retentate in the samples collected from mice administered free [^89^Zr]Zr^4+^ (Fig. 6C). Taken together, these data suggest that the radioplastics remained quite stable in vivo over the course of our investigation.

## Discussion

Micro- and nanoplastics have been found to be persistent and ubiquitous pollutants in a wide range of environments ranging from the predictable (soil, rivers, and the ocean) to the unexpected (indoor air, bottled water, and processed food)^1,5,7,12,14,15^. Yet despite this alarming threat, the bioaccumulation of micro- and nanoplastics in mammals has remained sorely understudied. We have attempted to close this knowledge gap by using PET imaging to track the in vivo fate of PS particles in mice after ingestion.

In pursuit of this goal, amine-bearing PS particles with four different diameters—20 nm, 220 nm, 1 μm, 6 μm—were used as scaffolds and modified with DFO and ^89^Zr. We chose ^89^Zr as our radionuclide due its widespread use in oncologic PET imaging. ^89^Zr is safe to use in animals and does not cause radiation-induced damage to tissues, especially when used in low doses as in this study. The [^89^Zr]Zr-DFO-PS radioplastics exhibited high stability in PBS, simulated gastric fluid, and simulated intestinal fluid over the course of several days, a time frame exceeding their biological residence time. Our chief concern during the construction of these radiolabeled particles was the extent to which the surface modifications would perturb their in vivo behavior compared to unmodified, pristine micro- and nanoplastics. Each of the [^89^Zr]Zr-DFO-PS particles synthesized, after all, was coated in amines, chelators, and radiometals rather than bare PS. Zeta potential measurements confirmed that the ^89^Zr-labeled radioplastics are not perfect recapitulations of unmodified PS particles, likely due to the positive charge that the amine group confers on their surface. The zeta potential of the plain PS particles hovered around − 85 mV compared to values between − 40 mV and − 50 mV for the NH_2_-PS, DFO-PS, and [^89^Zr]Zr-DFO-PS particles. This difference could, in theory, manifest in changes to the in vivo behavior of the particles. However, given the small size of the observed deviations, it is unlikely that the radioplastics would exhibit dramatically different physiochemical properties or pharmacokinetic profiles.

The in vivo experiments illustrated that the ingested [^89^Zr]Zr-DFO-PS particles remained stable in vivo, travelled through the gastrointestinal tract, and did not accumulate to any substantial degree in any other organ systems. Amongst the four radioplastics, the 20 nm [^89^Zr]Zr-DFO-PS particles travelled through the gastrointestinal tract most rapidly. Six hours after administration, the contents of the stomach contained only 7.0 ± 3.1% of the injected dose of the 20 nm particles compared to far higher amounts of the larger radioplastics: 34.6 ± 9.7%ID (220 nm), 40.3 ± 18.9%ID (1 µm), and 31.1 ± 9.7%ID (6 µm). At the same time point, the opposite trend was observed farther along the GI tract in the large intestines, where the contents contained 32.5 ± 9.5% of the injected dose of the 20 nm particles compared to 10.1 ± 4.5%, 8.6 ± 4.0%, and 5.6 ± 1.3% of the injected doses of the 220 nm, 1 µm, and 6 µm particles, respectively. This phenomenon is clearly visualized in the PET images, with the 20 nm particles delineating the lower GI tract far earlier than the three other radioplastics. While an exact reason for this behavior remains unknown, a likely explanation lies in the higher viscosity of the suspensions of the larger radioplastics.

The low activity concentrations of [^89^Zr]Zr-DFO-PS particles (*i.e.* < 1%ID/g) in all organs outside of the GI tract underscores the inability of the radioplastics to leave the gastrointestinal tract and reach systemic circulation in any substantial quantities. This is further reinforced by the absence of any substantial activity concentrations in the bone, a result of the mineralization of osteophilic [^89^Zr]Zr^4+^ that is frequently seen in conjunction with circulating ^89^Zr-labeled tracers such as radioimmunoconjugates^53^. Stock et al*.* and Rafiee et al*.* have likewise concluded that the translocation of PS microparticles after oral administration is minimal^32,33^. The European Food Safety Authority (EFSA) made a similar judgment in their 2016 report, noting that particles larger than 1.5 µm are too big to enter capillaries and cannot penetrate deep into organs^54,55^. Indeed, this work suggests that even the smallest [^89^Zr]Zr-DFO-PS particles (20 nm) do not cross the intestinal epithelial membrane in considerable quantities (empty stomach: 0.003%ID, empty SI: 0.001%ID, empty LI: 0.001%ID, and other organs: < 0.001%ID at 48 h after administration).

Yet these data nonetheless stand in contrast to previous studies with fluorophore-labeled PS particles administered via gavage that have found that the particles can reach systemic circulation and can thus be used as drug carriers, though with modest bioavailability at best^56–63^. In the majority of these investigations, the animals received daily doses of particles over the course of several days, and the total uptake of PS particles—as determined via fluorescence—varied between 3 to 38%^56–63^ (though in one case reached 100%^31^). We did not detect uptake anywhere near this high. Rather, our results for the intestinal walls are more similar to those reported by Walczak et al*.*^64^, who found 0.38–0.74% uptake 6 h after administration, data compared to our values of 0.10–0.36%ID. We did not, however, observe uptake levels in other organs (*e.g.* the kidneys, heart, and spleen) as high as those observed by Walczak et al.

The differences between the in vivo behavior observed in our study and some of these previous investigations could have several methodological explanations. For example, the dose of PS particles administered in our study was 25 times lower than that in the study by Walczak et al*.*^64^— ~ 5 mg/kg vs. ~ 125 mg/kg—a change which could clearly affect the pharmacokinetic profiles of the particles. We also employed a single dose compared to the several used by Florence et al*.*^56–63^ and Deng et al.^31^. Several toxicological studies have shown that continuous exposure to PS might have cumulative effects and cause disruptive cascade reactions in the body that could increase the ability of the particles to reach systemic circulation. Issues stemming from the previous investigators’ use of fluorophore-bearing PS particles may be in play as well. For example, the quantitative analysis of fluorescence imaging data can be clouded by self-quenching or the intrinsic auto-fluorescence of tissues^47^. Neither of these are concerns in the context of radioactivity detection. Even more worryingly—and unlike this study—none of the previous reports employing fluorophore-modified PS particles reported results from control cohorts that had been administered the fluorophore alone, raising the question of whether certain signals arose from free fluorophores or fluorescent PS particles. Finally, the attachment of bulky, hydrophobic fluorophores could influence the in vivo fate of these particles as well. Our investigation revealed that PS particles modified with amines, chelators, and radiometals had slightly altered zeta potentials compared to unmodified particles. The same would almost certainly be true—if not more dramatically so—for amine-decorated PS particles bearing bulky fluorophores. The pharmacokinetic impact of these perturbations is underscored by a systematic study of the biodistribution of fluorescent PS with varying surface charges that confirmed that several surface-related factors (*i.e.* not just the size) affected the bioavailability of the particles^64^.

## Conclusion

We contend that this investigation has illuminated the in vivo fate of micro- and nanoplastics after acute ingestion and—even more importantly—illustrated the efficacy of PET as a tool for the study of the pharmacokinetics and pharmacodynamics of plastic pollutants. Yet we believe that we have only scratched the surface of PET’s usefulness in this arena. Going forward, we plan to continue to develop these radioplastics and further leverage them to study the in vivo behavior of the ubiquitous plastic pollutants. With respect to the former, our primary goal is to develop radiolabeling methods for unmodified plastic particles in order to ensure that each radioplastic is an accurate recapitulation of the plastic particles in the environment. We will also expand the scope of radiolabeled micro- and nanoplastics to include other materials, including polyethylene (PE) and polypropylene (PP). Our future in vivo studies will explore the role of several variables in the pharmacokinetic profiles of micro- and nanoplastics, including the route of administration (*i.e.* ingestion vs. inhalation), the nature of exposure (*i.e.* acute vs. chronic), and the type of material (*i.e.* PS vs. PE vs PP). Ultimately, we aspire to create a body of work that helps unravel the public health threat of micro- and nanoplastics and establishes nuclear imaging as a critical component of the study of environmental pollutants of all kinds.

## Supplementary Information

Supplementary Information.

## Data Availability

The datasets generated during and/or analyzed during the current study are available from the corresponding authors upon reasonable request.

## Abbreviations

GI: Gastrointestinal
PdI: Polydispersity index
SGF: Simulated gastric fluid
SIF: Simulated intestinal fluid
FBP: Filtered back-projection
DMSO: Dimethyl sulfoxide
EDTA: Ethylenediaminetetraacetic acid
iTLC: Instant thin layer chromatography
EFSA: European food safety authority
PET: Positron emission tomography
DFO: Desferrioxamine
PS: Polystytere
PBS: Phosphate-buffered saline
%ID: Percent injected dose
%ID/g: Percent injected dose per gram of tissue
MIP: Maximum intensity projection
PE: Polyethylene
PP: Polypropylene

## References

[1] Gasperi J (2018). Microplastics in air: Are we breathing it in?. Curr. Opin. Environ. Sci. Health.

[2] Kontrick AV (2018). Microplastics and human health: Our great future to think about now. J. Med. Toxicol..

[3] Revel M, Châtel A, Mouneyrac C (2018). Micro(nano)plastics: A threat to human health?. Curr. Opin. Environ. Sci. Health.

[4] Wright SL, Kelly FJ (2017). Plastic and human health: A micro issue?. Environ. Sci. Technol..

[5] Alimba CG, Faggio C (2019). Microplastics in the marine environment: Current trends in environmental pollution and mechanisms of toxicological profile. Environ. Toxicol. Pharmacol..

[6] Ferreira I, Venâncio C, Lopes I, Oliveira M (2019). Nanoplastics and marine organisms: What has been studied?. Environ. Toxicol. Pharmacol..

[7] Koelmans AA (2019). Microplastics in freshwaters and drinking water: Critical review and assessment of data quality. Water Res..

[8] Rillig MC (2012). Microplastic in terrestrial ecosystems and the soil?. Environ. Sci. Technol..

[9] Rios Mendoza LM, Karapanagioti H, Álvarez NR (2018). Micro(nanoplastics) in the marine environment: Current knowledge and gaps. Curr. Opin. Environ. Sci. Health.

[10] Prata JC (2018). Airborne microplastics: Consequences to human health?. Environ. Pollut..

[11] Oßmann BE (2018). Small-sized microplastics and pigmented particles in bottled mineral water. Water Res..

[12] Peixoto D (2019). Microplastic pollution in commercial salt for human consumption: A review. Estuar. Coast. Shelf Sci..

[13] Pivokonsky M (2018). Occurrence of microplastics in raw and treated drinking water. Sci. Total Environ..

[14] Toussaint B (2019). Review of micro- and nanoplastic contamination in the food chain. Food Addit. Contam. Part A Chem Anal. Control. Expo. Risk Assess..

[15] Wang W, Gao H, Jin S, Li R, Na G (2019). The ecotoxicological effects of microplastics on aquatic food web, from primary producer to human: A review. Ecotoxicol. Environ. Saf..

[16] Schwabl P (2019). Detection of various microplastics in human stool: A prospective case series. Ann. Intern. Med..

[17] Ragusa A (2021). Plasticenta: First evidence of microplastics in human placenta. Environ. Int..

[18] Brun NR, Beenakker MMT, Hunting ER, Ebert D, Vijver MG (2017). Brood pouch-mediated polystyrene nanoparticle uptake during Daphnia magna embryogenesis. Nanotoxicology.

[19] Lin W (2019). Investigating the toxicities of different functionalized polystyrene nanoplastics on Daphnia magna. Ecotoxicol. Environ. Saf..

[20] Liu Z (2019). Polystyrene nanoplastic exposure induces immobilization, reproduction, and stress defense in the freshwater cladoceran Daphnia pulex. Chemosphere.

[21] Martins A, Guilhermino L (2018). Transgenerational effects and recovery of microplastics exposure in model populations of the freshwater cladoceran Daphnia magna Straus. Sci. Total Environ..

[22] Pitt JA (2018). Uptake, tissue distribution, and toxicity of polystyrene nanoparticles in developing zebrafish (Danio rerio). Aquat. Toxicol..

[23] Pitt JA (2018). Maternal transfer of nanoplastics to offspring in zebrafish (Danio rerio): A case study with nanopolystyrene. Sci. Total Environ..

[24] Qu M, Luo L, Yang Y, Kong Y, Wang D (2019). Nanopolystyrene-induced microRNAs response in *Caenorhabditis elegans* after long-term and lose-dose exposure. Sci. Total. Environ..

[25] Rochman CM, Kurobe T, Flores I, Teh SJ (2014). Early warning signs of endocrine disruption in adult fish from the ingestion of polyethylene with and without sorbed chemical pollutants from the marine environment. Sci. Total Environ..

[26] Tallec K (2018). Nanoplastics impaired oyster free living stages, gametes and embryos. Environ. Pollut..

[27] Shen M (2019). Recent advances in toxicological research of nanoplastics in the environment: A review. Environ. Pollut..

[28] Brun NR (2019). Polystyrene nanoplastics disrupt glucose metabolism and cortisol levels with a possible link to behavioural changes in larval zebrafish. Commun. Biol..

[29] Yong CQY, Valiyaveetill S, Tang BL (2020). Toxicity of Microplastics and Nanoplastics in Mammalian Systems. Int. J. Environ. Res. Public Health.

[30] Banerjee A, Shelver WL (2021). Micro- and nanoplastic induced cellular toxicity in mammals: A review. Sci. Total Environ..

[31] Deng Y, Zhang Y, Lemos B, Ren H (2017). Tissue accumulation of microplastics in mice and biomarker responses suggest widespread health risks of exposure. Sci. Rep..

[32] Stock V (2019). Uptake and effects of orally ingested polystyrene microplastic particles in vitro and in vivo. Arch. Toxicol..

[33] Rafiee M (2018). Neurobehavioral assessment of rats exposed to pristine polystyrene nanoplastics upon oral exposure. Chemosphere.

[34] Jin H (2021). Polystyrene microplastics induced male reproductive toxicity in mice. J. Hazard. Mater..

[35] Jin Y, Lu L, Tu W, Luo T, Fu Z (2019). Impacts of polystyrene microplastic on the gut barrier, microbiota and metabolism of mice. Sci. Total Environ..

[36] Lu L, Wan Z, Luo T, Fu Z, Jin Y (2018). Polystyrene microplastics induce gut microbiota dysbiosis and hepatic lipid metabolism disorder in mice. Sci. Total Environ..

[37] Yang Y-F, Chen C-Y, Lu T-H, Liao C-M (2019). Toxicity-based toxicokinetic/toxicodynamic assessment for bioaccumulation of polystyrene microplastics in mice. J. Hazard. Mater..

[38] Luo T (2019). Maternal polystyrene microplastic exposure during gestation and lactation altered metabolic homeostasis in the dams and their F1 and F2 offspring. Environ. Sci. Technol..

[39] Luo T (2019). Maternal exposure to different sizes of polystyrene microplastics during gestation causes metabolic disorders in their offspring. Environ. Poll..

[40] Li B (2020). Polyethylene microplastics affect the distribution of gut microbiota and inflammation development in mice. Chemosphere.

[41] Hou B, Wang F, Liu T, Wang Z (2020). Reproductive toxicity of polystyrene microplastics: In vivo experimental study on testicular toxicity in mice. J. Hazard Mater..

[42] Hu M, Palić D (2020). Micro- and nano-plastics activation of oxidative and inflammatory adverse outcome pathways. Redox. Biol..

[43] Zheng H, Wang J, Wei X, Chang L, Liu S (2021). Proinflammatory properties and lipid disturbance of polystyrene microplastics in the livers of mice with acute colitis. Sci. Total Environ..

[44] Fournier SB (2020). Nanopolystyrene translocation and fetal deposition after acute lung exposure during late-stage pregnancy. Part. Fibre Toxicol..

[45] Domenech J, Hernández A, Rubio L, Marcos R, Cortés C (2020). Interactions of polystyrene nanoplastics with in vitro models of the human intestinal barrier. Arch. Toxicol..

[46] Wu S, Wu M, Tian D, Qiu L, Li T (2020). Effects of polystyrene microbeads on cytotoxicity and transcriptomic profiles in human Caco-2 cells. Environ. Toxicol..

[47] Grafmueller S (2015). Transfer studies of polystyrene nanoparticles in the ex vivo human placenta perfusion model: Key sources of artifacts. Sci. Technol. Adv. Mater..

[48] Pietzonka P (2002). Transfer of lipophilic markers from PLGA and polystyrene nanoparticles to Caco-2 monolayers mimics particle uptake. Pharm. Res..

[49] Salvati A (2011). Experimental and theoretical comparison of intracellular import of polymeric nanoparticles and small molecules: toward models of uptake kinetics. Nanomed. Nanotechnol. Biol. Med..

[50] Tenuta T (2011). Elution of labile fluorescent dye from nanoparticles during biological use. PLoS ONE.

[51] Mahon E, Hristov DR, Dawson KA (2012). Stabilising fluorescent silica nanoparticles against dissolution effects for biological studies. Chem. Comm..

[52] Al-Sid-Cheikh M (2018). Uptake, whole-body distribution, and depuration of nanoplastics by the scallop pecten maximus at environmentally realistic concentrations. Environ. Sci. Technol..

[53] Abou DS, Ku T, Smith-Jones PM (2011). In vivo biodistribution and accumulation of 89Zr in mice. Nucl. Med. Biol..

[54] Yoo J-W, Doshi N, Mitragotri S (2011). Adaptive micro and nanoparticles: Temporal control over carrier properties to facilitate drug delivery. Adv. Drug Deliv. Rev..

[55] EFSA (2016). Presence of microplastics and nanoplastics in food, with particular focus on seafood. EFSA J..

[56] Florence AT, Hillery AM, Hussain N, Jani PU (1995). Factors affecting the oral uptake and translocation of polystyrene nanoparticles: Histological and analytical evidence. J. Drug. Target..

[57] Hillery AM, Florence AT (1996). The effect of adsorbed poloxamer 188 and 407 surfactants on the intestinal uptake of 60-nm polystyrene particles after oral administration in the rat. Int. J. Pharm..

[58] Hillery AM, Jani PU, Florence AT (1994). Comparative, quantitative study of lymphoid and non-lymphoid uptake of 60 nm polystyrene particles. J. Drug. Target..

[59] Jani P, Halbert GW, Langridge J, Florence AT (1990). Nanoparticle uptake by the rat gastrointestinal mucosa: Quantitation and particle size dependency. J. Pharm. Pharmacol..

[60] Jani P, Halbert GW, Langridge J, Florence AT (1989). The uptake and translocation of latex nanospheres and microspheres after oral administration to rats. J. Pharm. Pharmacol..

[61] Jani PU, Florence A, McCarthy DJ (1992). Further histological evidence of the gastrointestinal absorption of polystyrene nanospheres in the rat. Int. J. Pharm..

[62] Jani PU, McCarthy DE, Florence AT (1992). Nanosphere and microsphere uptake via Peyer's patches: Observation of the rate of uptake in the rat after a single oral dose. Int. J. Pharm..

[63] Jani PU (1996). Biliary excretion of polystyrene microspheres with covalently linked FITC fluorescence after oral and parenteral administration to male Wistar rats. J. Drug. Target..

[64] Walczak AP (2015). Bioavailability and biodistribution of differently charged polystyrene nanoparticles upon oral exposure in rats. J. Nanopart. Res..

