# Supplementary material for: Harnessing PET to track micro- and nanoplastics in vivo
Source: Sci Rep. 2021 Jun 1;11:11463. doi: 10.1038/s41598-021-90929-6 (PMC8169765; doi:10.1038/s41598-021-90929-6)
Supplement: Supplementary file 1 — Supplementary Information. [file 41598_2021_90929_MOESM1_ESM.pdf]

# SUPPORTING INFORMATION

## *Harnessing PET to Track Micro- and Nanoplastics In Vivo*

Outi Keinänen[1,2,3,*], Eric J. Dayts[1], Cindy Rodriguez[1,4], Samantha M. Sarrett[1,5], James M. Brennan[1], Mirkka Sarparanta[2], Brian M. Zeglis[1,3,4,5,6,*]

[1]Department of Chemistry, Hunter College, City University of New York, New York, NY, USA
[2]Department of Chemistry, University of Helsinki, Helsinki, Finland
[3]Department of Radiology, Memorial Sloan Kettering Cancer Center, New York, NY, USA
[4]Ph.D. Program in Chemistry, Graduate Center of the City University of New York, New York, NY, USA
[5]Ph.D. Program in Biochemistry, Graduate Center of the City University of New York, New York, NY, USA
[6]Department of Radiology, Weill Cornell Medical College, New York, NY, USA

***Co-Corresponding authors:** *Outi Keinänen and Brian M. Zeglis. 413 East 69th Street, New York, NY, 10021. Phone: 212-896-0433. Fax: 212-772-5332. E-mail: omk2009@med.cornell.edu and bz102@hunter.cuny.edu.

# Table of Contents


Table S1. *In vitro* stability data ........ 2
Figure S1. Transverse, coronal, and sagittal PET-CT slices ........ 3
Table S2. Biodistribution of [$^{89}$Zr]$Zr^{4+}$ (%ID/g) ........ 4
Table S3. Biodistribution of [$^{89}$Zr]$Zr^{4+}$ (%ID) ........ 5
Table S4. Biodistribution of [$^{89}$Zr]Zr-DFO (%ID/g) ........ 6
Table S5. Biodistribution of [$^{89}$Zr]Zr-DFO (%ID) ........ 7
Table S6. Biodistribution of [$^{89}$Zr]Zr-DFO-PS 20 nm (%ID/g) ........ 8
Table S7. Biodistribution of [$^{89}$Zr]Zr-DFO-PS 20 nm (%ID) ........ 9
Table S8. Biodistribution of [$^{89}$Zr]Zr-DFO-PS 220 nm (%ID/g) ........ 10
Table S9. Biodistribution of [$^{89}$Zr]Zr-DFO-PS 220 nm (%ID) ........ 11
Table S10. Biodistribution of [$^{89}$Zr]Zr-DFO-PS 1 µm (%ID/g) ........ 12
Table S11. Biodistribution of [$^{89}$Zr]Zr-DFO-PS 1 µm (%ID) ........ 13
Table S12. Biodistribution of [$^{89}$Zr]Zr-DFO-PS 6 µm (%ID/g) ........ 14
Table S13. Biodistribution of [$^{89}$Zr]Zr-DFO-PS 6 µm (%ID) ........ 15

**Table S1.** *In vitro* stability of [$^{89}$Zr]Zr-DFO-PS particles in phosphate buffered saline (PBS, pH 7.4), simulated gastric fluid (SGF, pH 3.0), and simulated intestinal fluid (SIF, pH 6.0). All experiments were performed in triplicate. The values are presented as mean ± standard deviation.

| | PBS | | | SGF | | | SIF | | |
|---|---|---|---|---|---|---|---|---|---|
| **20 nm, 24 h** | 99.5 | ± | 0.2 | 98.7 | ± | 0.4 | 100.0 | ± | 0.0 |
| **20 nm, 48 h** | 99.7 | ± | 0.1 | 98.8 | ± | 0.2 | 98.8 | ± | 2.0 |
| **20 nm, 96 h** | 99.2 | ± | 0.5 | 99.0 | ± | 0.2 | 94.0 | ± | 1.6 |
| **220 nm, 24 h** | 99.8 | ± | 0.1 | 99.4 | ± | 0.2 | 99.8 | ± | 0.2 |
| **220 nm, 48 h** | 99.8 | ± | 0.0 | 99.5 | ± | 0.2 | 99.8 | ± | 0.4 |
| **220 nm, 96 h** | 99.4 | ± | 0.2 | 99.6 | ± | 0.4 | 99.3 | ± | 0.5 |
| **1 µm, 24 h** | 99.9 | ± | 0.2 | 98.2 | ± | 0.4 | 99.7 | ± | 0.0 |
| **1 µm, 48 h** | 99.9 | ± | 0.1 | 97.4 | ± | 0.3 | 100.0 | ± | 0.0 |
| **1 µm, 96 h** | 100.0 | ± | 0.0 | 99.2 | ± | 0.2 | 98.6 | ± | 0.1 |
| **6 µm, 24 h** | 99.7 | ± | 0.1 | 98.7 | ± | 0.1 | 99.9 | ± | 0.1 |
| **6 µm, 48 h** | 99.8 | ± | 0.0 | 99.1 | ± | 0.1 | 99.9 | ± | 0.1 |
| **6 µm, 96 h** | 100.0 | ± | 0.0 | 99.2 | ± | 0.0 | 99.8 | ± | 0.1 |

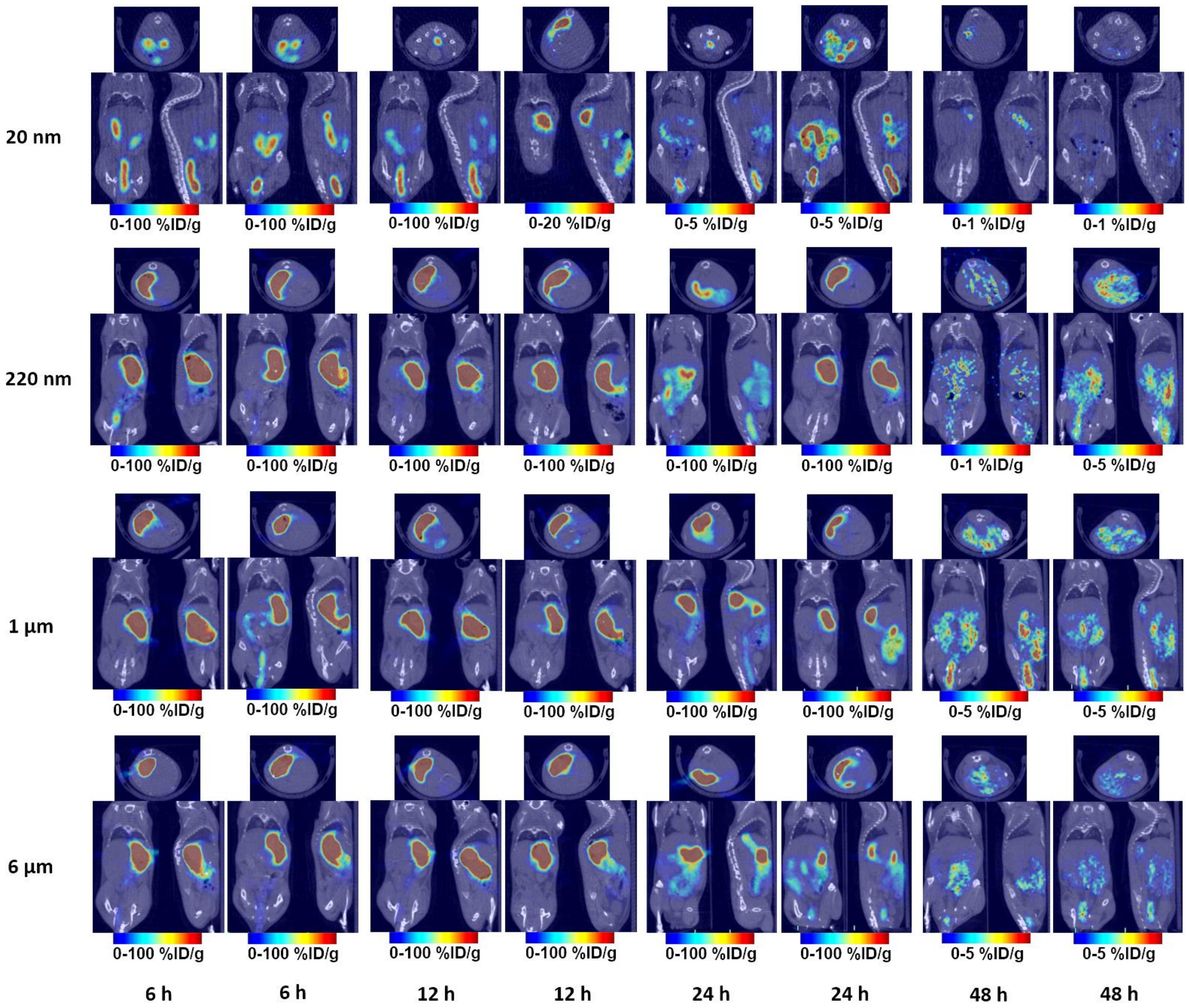


**Figure S1.** Transverse, coronal, and sagittal PET-CT slices of mice that received 1.85 MBq (50 µCi) of [$^{89}$Zr]Zr-DFO-PS *per os*.

**Table S2.** Biodistribution of [$^{89}$Zr]Zr$^{4+}$ (1.85 MBq in 0.1 mL of PBS) after oral administration via gavage to healthy, immunocompetent mice. Values are in units of %ID/g and are expressed as mean ± standard deviation.

| **%ID/g (percentage of injected dose per gram of organ)** | | | | |
|---|---|---|---|---|
| **Organ** | 6 h | 12 h | 24 h | 48 h |
| **Urine** | 0.0243 ± 0.0154 | 0.0081 ± 0.0067 | 0.0072 ± 0.0067 | 0.0218 ± 0.0257 |
| **Blood** | 0.0033 ± 0.0032 | 0.0021 ± 0.0006 | 0.0010 ± 0.0006 | 0.0002 ± 0.0002 |
| **Gallbladder** | 0.4899 ± 0.3173 | 0.2730 ± 0.0626 | 0.0630 ± 0.0595 | 0.0015 ± 0.0021 |
| **Heart** | 0.0040 ± 0.0023 | 0.0033 ± 0.0041 | 0.0082 ± 0.0153 | 0.0001 ± 0.0002 |
| **Lungs** | 0.0019 ± 0.0017 | 0.0013 ± 0.0017 | 0.0012 ± 0.0009 | 0.0006 ± 0.0007 |
| **Liver** | 0.0009 ± 0.0007 | 0.0006 ± 0.0003 | 0.0005 ± 0.0004 | 0.0002 ± 0.0001 |
| **Spleen** | 0.0037 ± 0.0012 | 0.0021 ± 0.0032 | 0.0057 ± 0.0057 | 0.0009 ± 0.0010 |
| **Pancreas** | 0.0025 ± 0.0015 | 0.0006 ± 0.0009 | 0.0035 ± 0.0027 | 0.0002 ± 0.0003 |
| **Kidneys** | 0.0016 ± 0.0014 | 0.0011 ± 0.0008 | 0.0021 ± 0.0011 | 0.0009 ± 0.0006 |
| **Ovaries** | 0.0016 ± 0.0007 | 0.0006 ± 0.0005 | 0.0013 ± 0.0006 | 0.0003 ± 0.0003 |
| **Bone (femur)** | 0.0133 ± 0.0062 | 0.0090 ± 0.0202 | 0.0136 ± 0.0107 | 0.0037 ± 0.0043 |
| **Muscle** | 0.0022 ± 0.0020 | 0.0015 ± 0.0013 | 0.0012 ± 0.0014 | 0.0007 ± 0.0006 |
| **Stomach (empty)** | 4.822 ± 2.966 | 0.278 ± 0.531 | 0.063 ± 0.024 | 0.023 ± 0.012 |
| **Stomach contents** | 37.563 ± 28.257 | 0.566 ± 1.192 | 2.168 ± 0.939 | 0.308 ± 0.160 |
| **S. intestine (empty)** | 1.160 ± 0.842 | 0.089 ± 0.185 | 0.018 ± 0.007 | 0.004 ± 0.002 |
| **S. intestine contents** | 31.197 ± 28.567 | 4.004 ± 8.751 | 0.564 ± 0.325 | 0.053 ± 0.013 |
| **L. intestine (empty)** | 1.807 ± 1.743 | 1.036 ± 0.638 | 0.088 ± 0.027 | 0.016 ± 0.012 |
| **L. intestine contents** | 23.981 ± 8.656 | 17.609 ± 11.885 | 2.620 ± 1.595 | 0.216 ± 0.102 |

**Table S3.** Biodistribution of [$^{89}$Zr]Zr$^{4+}$ (1.85 MBq in 0.1 mL of PBS) after oral administration via gavage to healthy, immunocompetent mice. Values are in units of %ID and are expressed as mean ± standard deviation.

| | **%ID (percentage of injected dose)** | | | |
|---|---|---|---|---|
| **Organ** | 6 h | 12 h | 24 h | 48 h |
| **Urine** | 0.00112 ± 0.00069 | 0.00026 ± 0.00026 | 0.00028 ± 0.00027 | 0.00010 ± 0.00000 |
| **Blood** | 0.00128 ± 0.00177 | 0.00064 ± 0.00011 | 0.00024 ± 0.00019 | 0.00010 ± 0.00007 |
| **Gallbladder** | 0.00014 ± 0.00011 | 0.00010 ± 0.00014 | 0.00012 ± 0.00013 | 0.00000 ± 0.00000 |
| **Heart** | 0.00026 ± 0.00017 | 0.00028 ± 0.00035 | 0.00058 ± 0.00108 | 0.00000 ± 0.00000 |
| **Lungs** | 0.00026 ± 0.00022 | 0.00016 ± 0.00023 | 0.00020 ± 0.00016 | 0.00010 ± 0.00014 |
| **Liver** | 0.00098 ± 0.00066 | 0.00064 ± 0.00027 | 0.00056 ± 0.00040 | 0.00022 ± 0.00013 |
| **Spleen** | 0.00016 ± 0.00005 | 0.00008 ± 0.00013 | 0.00020 ± 0.00014 | 0.00004 ± 0.00005 |
| **Pancreas** | 0.00022 ± 0.00016 | 0.00004 ± 0.00005 | 0.00038 ± 0.00029 | 0.00002 ± 0.00004 |
| **Kidneys** | 0.00040 ± 0.00034 | 0.00030 ± 0.00020 | 0.00054 ± 0.00026 | 0.00024 ± 0.00015 |
| **Ovaries** | 0.00028 ± 0.00013 | 0.00008 ± 0.00008 | 0.00022 ± 0.00011 | 0.00004 ± 0.00005 |
| **Bone (femur)** | 0.00016 ± 0.00009 | 0.00008 ± 0.00018 | 0.00018 ± 0.00015 | 0.00004 ± 0.00009 |
| **Muscle** | 0.00016 ± 0.00013 | 0.00014 ± 0.00011 | 0.00010 ± 0.00010 | 0.00006 ± 0.00005 |
| **Stomach (empty)** | 0.559 ± 0.306 | 0.029 ± 0.056 | 0.007 ± 0.004 | 0.003 ± 0.002 |
| **Stomach contents** | 21.778 ± 13.574 | 0.440 ± 0.935 | 0.506 ± 0.211 | 0.049 ± 0.027 |
| **S. intestine (empty)** | 0.724 ± 0.498 | 0.048 ± 0.100 | 0.008 ± 0.003 | 0.002 ± 0.001 |
| **S. intestine contents** | 14.630 ± 12.287 | 2.293 ± 5.017 | 0.348 ± 0.175 | 0.036 ± 0.010 |
| **L. intestine (empty)** | 0.469 ± 0.455 | 0.258 ± 0.140 | 0.020 ± 0.006 | 0.004 ± 0.002 |
| **L. intestine contents** | 9.824 ± 3.591 | 6.430 ± 4.825 | 1.353 ± 0.723 | 0.128 ± 0.070 |

**Table S4.** Biodistribution of [$^{89}$Zr]Zr-DFO (1.85 MBq, 0.1 mg, in 0.1 ml of PBS) after oral administration via gavage to healthy, immunocompetent mice. Values are in units of %ID/g and are expressed as mean ± standard deviation.

| %ID/g (percentage of injected dose per gram of organ) | | | | | | | | | | | | |
|---|---|---|---|---|---|---|---|---|---|---|---|---|
| **Organ** | 6 h | | | 12 h | | | 24 h | | | 48 h | | |
| **Urine** | 0.0142 | ± | 0.0115 | 0.0146 | ± | 0.0115 | 0.0172 | ± | 0.0255 | 0.1161 | ± | 0.0853 |
| **Blood** | 0.0005 | ± | 0.0004 | 0.0011 | ± | 0.0002 | 0.0010 | ± | 0.0008 | 0.0019 | ± | 0.0014 |
| **Gallbladder** | 0.2493 | ± | 0.0228 | 0.3267 | ± | 0.2295 | 0.1794 | ± | 0.2471 | 0.1825 | ± | 0.1713 |
| **Heart** | 0.0011 | ± | 0.0019 | 0.0054 | ± | 0.0021 | 0.0054 | ± | 0.0031 | 0.0090 | ± | 0.0070 |
| **Lungs** | 0.0007 | ± | 0.0010 | 0.0021 | ± | 0.0011 | 0.0018 | ± | 0.0013 | 0.0039 | ± | 0.0035 |
| **Liver** | 0.0006 | ± | 0.0005 | 0.0005 | ± | 0.0004 | 0.0002 | ± | 0.0001 | 0.0009 | ± | 0.0005 |
| **Spleen** | 0.0021 | ± | 0.0017 | 0.0054 | ± | 0.0036 | 0.0045 | ± | 0.0052 | 0.0144 | ± | 0.0112 |
| **Pancreas** | 0.0010 | ± | 0.0016 | 0.0012 | ± | 0.0007 | 0.0027 | ± | 0.0024 | 0.0062 | ± | 0.0052 |
| **Kidneys** | 0.0019 | ± | 0.0012 | 0.0014 | ± | 0.0004 | 0.0027 | ± | 0.0012 | 0.0037 | ± | 0.0027 |
| **Ovaries** | 0.0007 | ± | 0.0013 | 0.0037 | ± | 0.0032 | 0.0013 | ± | 0.0006 | 0.0063 | ± | 0.0044 |
| **Bone (femur)** | 0.0086 | ± | 0.0159 | 0.0226 | ± | 0.0158 | 0.0121 | ± | 0.0169 | 0.0944 | ± | 0.0448 |
| **Muscle** | 0.0020 | ± | 0.0027 | 0.0034 | ± | 0.0012 | 0.0015 | ± | 0.0014 | 0.0103 | ± | 0.0074 |
| **Stomach (empty)** | 7.22 | ± | 11.08 | 0.14 | ± | 0.10 | 0.06 | ± | 0.04 | 0.07 | ± | 0.10 |
| **Stomach contents** | 21.58 | ± | 29.66 | 1.61 | ± | 1.55 | 2.04 | ± | 0.94 | 0.92 | ± | 1.29 |
| **S. intestine (empty)** | 1.26 | ± | 0.88 | 0.11 | ± | 0.09 | 0.05 | ± | 0.02 | 0.01 | ± | 0.01 |
| **S. intestine contents** | 36.59 | ± | 27.07 | 3.89 | ± | 3.56 | 1.08 | ± | 0.57 | 0.28 | ± | 0.46 |
| **L. intestine (empty)** | 1.12 | ± | 0.74 | 0.54 | ± | 0.23 | 0.11 | ± | 0.05 | 0.04 | ± | 0.03 |
| **L. intestine contents** | 31.75 | ± | 15.29 | 16.41 | ± | 6.23 | 2.03 | ± | 2.15 | 0.50 | ± | 0.72 |

**Table S5.** Biodistribution of [$^{89}$Zr]Zr-DFO (1.85 MBq, 0.1 mg, in 0.1 ml of PBS) after oral administration via gavage to healthy, immunocompetent mice. Values are in units of %ID and are expressed as mean ± standard deviation.

| | %ID (percentage of injected dose) | | | | | | | | | | | |
|---|---|---|---|---|---|---|---|---|---|---|---|---|
| **Organ** | 6 h | | | 12 h | | | 24 h | | | 48 h | | |
| **Urine** | 0.00012 | ± | 0.00018 | 0.00028 | ± | 0.00016 | 0.00032 | ± | 0.00022 | 0.00100 | ± | 0.00035 |
| **Blood** | 0.00026 | ± | 0.00030 | 0.00036 | ± | 0.00009 | 0.00022 | ± | 0.00018 | 0.00086 | ± | 0.00072 |
| **Gallbladder** | 0.00012 | ± | 0.00011 | 0.00018 | ± | 0.00011 | 0.00032 | ± | 0.00022 | 0.00090 | ± | 0.00051 |
| **Heart** | 0.00008 | ± | 0.00013 | 0.00036 | ± | 0.00011 | 0.00046 | ± | 0.00029 | 0.00076 | ± | 0.00060 |
| **Lungs** | 0.00006 | ± | 0.00009 | 0.00030 | ± | 0.00020 | 0.00030 | ± | 0.00023 | 0.00076 | ± | 0.00069 |
| **Liver** | 0.00060 | ± | 0.00057 | 0.00064 | ± | 0.00054 | 0.00034 | ± | 0.00023 | 0.00098 | ± | 0.00058 |
| **Spleen** | 0.00008 | ± | 0.00008 | 0.00028 | ± | 0.00018 | 0.00026 | ± | 0.00033 | 0.00094 | ± | 0.00071 |
| **Pancreas** | 0.00008 | ± | 0.00013 | 0.00012 | ± | 0.00011 | 0.00032 | ± | 0.00025 | 0.00088 | ± | 0.00075 |
| **Kidneys** | 0.00046 | ± | 0.00026 | 0.00038 | ± | 0.00013 | 0.00074 | ± | 0.00034 | 0.00106 | ± | 0.00074 |
| **Ovaries** | 0.00012 | ± | 0.00022 | 0.00042 | ± | 0.00033 | 0.00020 | ± | 0.00007 | 0.00104 | ± | 0.00065 |
| **Bone (femur)** | 0.00014 | ± | 0.00026 | 0.00024 | ± | 0.00017 | 0.00012 | ± | 0.00022 | 0.00122 | ± | 0.00051 |
| **Muscle** | 0.00014 | ± | 0.00017 | 0.00032 | ± | 0.00011 | 0.00010 | ± | 0.00019 | 0.00104 | ± | 0.00075 |
| **Stomach (empty)** | 0.881 | ± | 1.364 | 0.017 | ± | 0.013 | 0.007 | ± | 0.005 | 0.009 | ± | 0.014 |
| **Stomach contents** | 12.218 | ± | 15.604 | 1.177 | ± | 1.078 | 0.615 | ± | 0.316 | 0.124 | ± | 0.203 |
| **S. intestine (empty)** | 0.849 | ± | 0.628 | 0.068 | ± | 0.058 | 0.020 | ± | 0.010 | 0.004 | ± | 0.004 |
| **S. intestine contents** | 17.633 | ± | 12.010 | 2.427 | ± | 2.197 | 0.739 | ± | 0.424 | 0.174 | ± | 0.281 |
| **L. intestine (empty)** | 0.258 | ± | 0.149 | 0.136 | ± | 0.063 | 0.026 | ± | 0.013 | 0.013 | ± | 0.010 |
| **L. intestine contents** | 11.514 | ± | 5.517 | 6.734 | ± | 1.879 | 1.327 | ± | 1.458 | 0.295 | ± | 0.425 |

**Table S6.** Biodistribution of 20 nm [$^{89}$Zr]Zr-DFO-PS particles (1.85 MBq, 0.1 mg, in 0.1 ml of PBS-T) after oral administration via gavage to healthy, immunocompetent mice. Values are in units of %ID/g and are expressed as mean ± standard deviation.

| %ID/g (percentage of injected dose per gram of organ) | | | | | | | | | | | | |
|---|---|---|---|---|---|---|---|---|---|---|---|---|
| **Organ** | 6 h | | | 12 h | | | 24 h | | | 48 h | | |
| **Urine** | 0.08706 | ± | 0.11695 | 0.02026 | ± | 0.00968 | 0.00197 | ± | 0.00340 | 0.01144 | ± | 0.00607 |
| **Blood** | 0.00343 | ± | 0.00191 | 0.00242 | ± | 0.00045 | 0.00036 | ± | 0.00011 | 0.00239 | ± | 0.00152 |
| **Gallbladder** | 0.12599 | ± | 0.11148 | 0.00963 | ± | 0.01349 | 0.04437 | ± | 0.04947 | 0.28098 | ± | 0.27358 |
| **Heart** | 0.00117 | ± | 0.00114 | 0.00108 | ± | 0.00094 | 0.00141 | ± | 0.00100 | 0.00583 | ± | 0.00360 |
| **Lungs** | 0.00328 | ± | 0.00180 | 0.00077 | ± | 0.00059 | 0.00057 | ± | 0.00040 | 0.00289 | ± | 0.00103 |
| **Liver** | 0.00145 | ± | 0.00157 | 0.00068 | ± | 0.00021 | 0.00021 | ± | 0.00014 | 0.00082 | ± | 0.00043 |
| **Spleen** | 0.00205 | ± | 0.00180 | 0.00093 | ± | 0.00207 | 0.00152 | ± | 0.00154 | 0.00969 | ± | 0.00247 |
| **Pancreas** | 0.00455 | ± | 0.00907 | 0.00057 | ± | 0.00065 | 0.00039 | ± | 0.00031 | 0.00414 | ± | 0.00132 |
| **Kidneys** | 0.00171 | ± | 0.00104 | 0.00180 | ± | 0.00050 | 0.00073 | ± | 0.00045 | 0.00278 | ± | 0.00098 |
| **Ovaries** | 0.00066 | ± | 0.00045 | 0.00065 | ± | 0.00058 | 0.00070 | ± | 0.00039 | 0.00295 | ± | 0.00040 |
| **Bone (femur)** | 0.00320 | ± | 0.00440 | 0.00304 | ± | 0.00394 | 0.00641 | ± | 0.00556 | 0.04741 | ± | 0.01626 |
| **Muscle** | 0.00104 | ± | 0.00086 | 0.00036 | ± | 0.00067 | 0.00198 | ± | 0.00165 | 0.00712 | ± | 0.00161 |
| **Stomach (empty)** | 1.298 | ± | 0.416 | 0.235 | ± | 0.168 | 0.021 | ± | 0.011 | 0.020 | ± | 0.008 |
| **Stomach contents** | 70.914 | ± | 52.458 | 7.454 | ± | 6.294 | 0.861 | ± | 1.118 | 0.085 | ± | 0.096 |
| **S. intestine (empty)** | 0.837 | ± | 0.452 | 0.053 | ± | 0.035 | 0.007 | ± | 0.006 | 0.002 | ± | 0.001 |
| **S. intestine contents** | 24.603 | ± | 17.761 | 2.111 | ± | 1.853 | 0.272 | ± | 0.189 | 0.018 | ± | 0.014 |
| **L. intestine (empty)** | 2.270 | ± | 1.063 | 0.513 | ± | 0.346 | 0.034 | ± | 0.030 | 0.005 | ± | 0.002 |
| **L. intestine contents** | 89.429 | ± | 29.267 | 19.526 | ± | 13.571 | 1.546 | ± | 1.796 | 0.045 | ± | 0.029 |

**Table S7.** Biodistribution of 20 nm [$^{89}$Zr]Zr-DFO-PS particles (1.85 MBq, 0.1 mg, in 0.1 ml of PBS-T) after oral administration via gavage to healthy, immunocompetent mice. Values are in units of %ID and are expressed as mean ± standard deviation.

| | %ID (percentage of injected dose) | | | | | | | | | | | |
|---|---|---|---|---|---|---|---|---|---|---|---|---|
| **Organ** | 6 h | | | 12 h | | | 24 h | | | 48 h | | |
| **Urine** | 0.00109 | ± | 0.00135 | 0.00061 | ± | 0.00087 | 0.00007 | ± | 0.00011 | 0.00047 | ± | 0.00021 |
| **Blood** | 0.00083 | ± | 0.00037 | 0.00047 | ± | 0.00021 | 0.00018 | ± | 0.00008 | 0.00051 | ± | 0.00018 |
| **Gallbladder** | 0.00048 | ± | 0.00038 | 0.00006 | ± | 0.00000 | 0.00011 | ± | 0.00009 | 0.00048 | ± | 0.00018 |
| **Heart** | 0.00007 | ± | 0.00007 | 0.00008 | ± | 0.00006 | 0.00011 | ± | 0.00009 | 0.00052 | ± | 0.00032 |
| **Lungs** | 0.00046 | ± | 0.00026 | 0.00012 | ± | 0.00009 | 0.00010 | ± | 0.00007 | 0.00048 | ± | 0.00018 |
| **Liver** | 0.00106 | ± | 0.00099 | 0.00060 | ± | 0.00015 | 0.00022 | ± | 0.00014 | 0.00078 | ± | 0.00032 |
| **Spleen** | 0.00007 | ± | 0.00006 | 0.00004 | ± | 0.00008 | 0.00009 | ± | 0.00010 | 0.00054 | ± | 0.00023 |
| **Pancreas** | 0.00043 | ± | 0.00087 | 0.00006 | ± | 0.00007 | 0.00006 | ± | 0.00005 | 0.00055 | ± | 0.00019 |
| **Kidneys** | 0.00040 | ± | 0.00022 | 0.00050 | ± | 0.00015 | 0.00024 | ± | 0.00016 | 0.00082 | ± | 0.00035 |
| **Ovaries** | 0.00011 | ± | 0.00010 | 0.00012 | ± | 0.00012 | 0.00012 | ± | 0.00007 | 0.00053 | ± | 0.00012 |
| **Bone (femur)** | 0.00004 | ± | 0.00006 | 0.00004 | ± | 0.00005 | 0.00010 | ± | 0.00009 | 0.00054 | ± | 0.00012 |
| **Muscle** | 0.00007 | ± | 0.00005 | 0.00003 | ± | 0.00005 | 0.00014 | ± | 0.00011 | 0.00059 | ± | 0.00011 |
| **Stomach (empty)** | 0.117 | ± | 0.029 | 0.026 | ± | 0.017 | 0.003 | ± | 0.002 | 0.003 | ± | 0.001 |
| **Stomach contents** | 7.024 | ± | 3.189 | 4.149 | ± | 2.897 | 0.261 | ± | 0.414 | 0.015 | ± | 0.014 |
| **S. intestine (empty)** | 0.276 | ± | 0.149 | 0.028 | ± | 0.015 | 0.004 | ± | 0.004 | 0.001 | ± | 0.001 |
| **S. intestine contents** | 8.288 | ± | 5.279 | 1.077 | ± | 0.877 | 0.129 | ± | 0.087 | 0.007 | ± | 0.006 |
| **L. intestine (empty)** | 0.344 | ± | 0.125 | 0.118 | ± | 0.077 | 0.009 | ± | 0.008 | 0.001 | ± | 0.001 |
| **L. intestine contents** | 32.484 | ± | 9.471 | 6.190 | ± | 4.166 | 0.990 | ± | 1.168 | 0.027 | ± | 0.017 |

**Table S8.** Biodistribution of 220 nm [$^{89}$Zr]Zr-DFO-PS particles (1.85 MBq. 0.1 mg. in 0.1 ml of PBS) after oral administration via gavage to healthy, immunocompetent mice. Values are in units of %ID/g and are expressed as mean ± standard deviation.

| %ID/g (percentage of injected dose per gram of organ) | | | | | | | | | | | | |
|---|---|---|---|---|---|---|---|---|---|---|---|---|
| **Organ** | | 6 h | | | 12 h | | | 24 h | | | 48 h | |
| **Urine** | 0.0208 | ± | 0.0187 | 0.0064 | ± | 0.0041 | 0.0291 | ± | 0.0195 | 0.2954 | ± | 0.1100 |
| **Blood** | 0.0011 | ± | 0.0009 | 0.0007 | ± | 0.0006 | 0.0043 | ± | 0.0036 | 0.0012 | ± | 0.0013 |
| **Gallbladder** | 0.1676 | ± | 0.1694 | 0.0656 | ± | 0.0785 | 0.2336 | ± | 0.3529 | 0.6552 | ± | 0.5863 |
| **Heart** | 0.0017 | ± | 0.0013 | 0.0012 | ± | 0.0008 | 0.0091 | ± | 0.0046 | 0.0046 | ± | 0.0048 |
| **Lungs** | 0.0017 | ± | 0.0015 | 0.0012 | ± | 0.0005 | 0.0036 | ± | 0.0020 | 0.0025 | ± | 0.0020 |
| **Liver** | 0.0009 | ± | 0.0007 | 0.0003 | ± | 0.0003 | 0.0012 | ± | 0.0015 | 0.0007 | ± | 0.0003 |
| **Spleen** | 0.0020 | ± | 0.0011 | 0.0015 | ± | 0.0006 | 0.0090 | ± | 0.0070 | 0.0103 | ± | 0.0096 |
| **Pancreas** | 0.0026 | ± | 0.0008 | 0.0022 | ± | 0.0010 | 0.0058 | ± | 0.0046 | 0.0044 | ± | 0.0045 |
| **Kidneys** | 0.0012 | ± | 0.0006 | 0.0012 | ± | 0.0010 | 0.0038 | ± | 0.0035 | 0.0022 | ± | 0.0021 |
| **Ovaries** | 0.0020 | ± | 0.0013 | 0.0011 | ± | 0.0007 | 0.0048 | ± | 0.0018 | 0.0038 | ± | 0.0026 |
| **Bone (femur)** | 0.0129 | ± | 0.0167 | 0.0213 | ± | 0.0262 | 0.0435 | ± | 0.0226 | 0.0473 | ± | 0.0360 |
| **Muscle** | 0.0027 | ± | 0.0010 | 0.0064 | ± | 0.0096 | 0.0067 | ± | 0.0049 | 0.0070 | ± | 0.0060 |
| **Stomach (empty)** | 3.214 | ± | 2.524 | 0.232 | ± | 0.168 | 0.024 | ± | 0.010 | 0.057 | ± | 0.029 |
| **Stomach contents** | 45.334 | ± | 16.381 | 9.822 | ± | 12.305 | 0.304 | ± | 0.305 | 0.753 | ± | 0.768 |
| **S. intestine (empty)** | 0.465 | ± | 0.308 | 0.241 | ± | 0.160 | 0.004 | ± | 0.002 | 0.006 | ± | 0.003 |
| **S. intestine contents** | 20.210 | ± | 13.361 | 7.648 | ± | 6.081 | 0.094 | ± | 0.121 | 0.292 | ± | 0.359 |
| **L. intestine (empty)** | 0.445 | ± | 0.364 | 0.465 | ± | 0.208 | 0.027 | ± | 0.026 | 0.027 | ± | 0.022 |
| **L. intestine contents** | 21.479 | ± | 9.621 | 17.361 | ± | 10.740 | 0.274 | ± | 0.277 | 0.473 | ± | 0.529 |

**Table S9.** Biodistribution of 220 nm [$^{89}$Zr]Zr-DFO-PS particles (1.85 MBq. 0.1 mg. in 0.1 ml of PBS) after oral administration via gavage to healthy, immunocompetent mice. Values are in units of %ID and are expressed as mean ± standard deviation.

| | **%ID (percentage of injected dose)** | | | | | | | | | | | |
|---|---|---|---|---|---|---|---|---|---|---|---|---|
| **Organ** | 6 h | | | 12 h | | | 24 h | | | 48 h | | |
| **Urine** | 0.00048 | ± | 0.00015 | 0.00023 | ± | 0.00010 | 0.00078 | ± | 0.00055 | 0.00063 | ± | 0.00012 |
| **Blood** | 0.00022 | ± | 0.00004 | 0.00014 | ± | 0.00011 | 0.00110 | ± | 0.00086 | 0.00044 | ± | 0.00052 |
| **Gallbladder** | 0.00010 | ± | 0.00000 | 0.00010 | ± | 0.00000 | 0.00082 | ± | 0.00036 | 0.00047 | ± | 0.00006 |
| **Heart** | 0.00018 | ± | 0.00015 | 0.00012 | ± | 0.00008 | 0.00084 | ± | 0.00041 | 0.00032 | ± | 0.00033 |
| **Lungs** | 0.00024 | ± | 0.00023 | 0.00018 | ± | 0.00008 | 0.00063 | ± | 0.00033 | 0.00036 | ± | 0.00023 |
| **Liver** | 0.00100 | ± | 0.00086 | 0.00028 | ± | 0.00020 | 0.00148 | ± | 0.00191 | 0.00072 | ± | 0.00038 |
| **Spleen** | 0.00016 | ± | 0.00009 | 0.00010 | ± | 0.00007 | 0.00058 | ± | 0.00050 | 0.00032 | ± | 0.00039 |
| **Pancreas** | 0.00020 | ± | 0.00007 | 0.00024 | ± | 0.00011 | 0.00070 | ± | 0.00056 | 0.00036 | ± | 0.00040 |
| **Kidneys** | 0.00036 | ± | 0.00021 | 0.00036 | ± | 0.00031 | 0.00112 | ± | 0.00102 | 0.00056 | ± | 0.00048 |
| **Ovaries** | 0.00020 | ± | 0.00000 | 0.00018 | ± | 0.00013 | 0.00072 | ± | 0.00028 | 0.00058 | ± | 0.00036 |
| **Bone (femur)** | 0.00010 | ± | 0.00007 | 0.00010 | ± | 0.00007 | 0.00068 | ± | 0.00030 | 0.00068 | ± | 0.00039 |
| **Muscle** | 0.00022 | ± | 0.00008 | 0.00040 | ± | 0.00057 | 0.00070 | ± | 0.00048 | 0.00052 | ± | 0.00044 |
| **Stomach (empty)** | 0.432 | ± | 0.369 | 0.030 | ± | 0.024 | 0.003 | ± | 0.001 | 0.005 | ± | 0.003 |
| **Stomach contents** | 34.627 | ± | 9.688 | 5.899 | ± | 7.489 | 0.055 | ± | 0.055 | 0.143 | ± | 0.157 |
| **S. intestine (empty)** | 0.170 | ± | 0.118 | 0.105 | ± | 0.073 | 0.002 | ± | 0.001 | 0.003 | ± | 0.002 |
| **S. intestine contents** | 13.203 | ± | 7.375 | 4.704 | ± | 3.944 | 0.053 | ± | 0.073 | 0.124 | ± | 0.189 |
| **L. intestine (empty)** | 0.091 | ± | 0.077 | 0.105 | ± | 0.054 | 0.007 | ± | 0.007 | 0.007 | ± | 0.006 |
| **L. intestine contents** | 10.111 | ± | 4.501 | 7.707 | ± | 4.621 | 0.165 | ± | 0.171 | 0.261 | ± | 0.314 |

**Table S10.** Biodistribution of 1 µm [$^{89}$Zr]Zr-DFO-PS particles (1.85 MBq. 0.1 mg. in 0.1 ml of PBS) after oral administration via gavage to healthy, immunocompetent mice. Values are in units of %ID/g and are expressed as mean ± standard deviation.

| %ID/g (percentage of injected dose per gram of organ) | | | | | | | | | | | | |
|---|---|---|---|---|---|---|---|---|---|---|---|---|
| **Organ** | 6 h | | | 12 h | | | 24 h | | | 48 h | | |
| **Urine** | 0.0199 | ± | 0.0045 | 0.0064 | ± | 0.0032 | 0.0286 | ± | 0.0264 | 0.0598 | ± | 0.0055 |
| **Blood** | 0.0009 | ± | 0.0006 | 0.0011 | ± | 0.0009 | 0.0005 | ± | 0.0009 | 0.0011 | ± | 0.0014 |
| **Gallbladder** | 0.0995 | ± | 0.0421 | 0.1185 | ± | 0.1170 | 0.2846 | ± | 0.4973 | 0.3498 | ± | 0.2401 |
| **Heart** | 0.0008 | ± | 0.0011 | 0.0015 | ± | 0.0006 | 0.0033 | ± | 0.0068 | 0.0057 | ± | 0.0079 |
| **Lungs** | 0.0012 | ± | 0.0010 | 0.0011 | ± | 0.0006 | 0.0015 | ± | 0.0028 | 0.0018 | ± | 0.0024 |
| **Liver** | 0.0039 | ± | 0.0045 | 0.0003 | ± | 0.0001 | 0.0004 | ± | 0.0004 | 0.0003 | ± | 0.0006 |
| **Spleen** | 0.0026 | ± | 0.0017 | 0.0022 | ± | 0.0012 | 0.0073 | ± | 0.0113 | 0.0110 | ± | 0.0166 |
| **Pancreas** | 0.0033 | ± | 0.0050 | 0.0012 | ± | 0.0018 | 0.0025 | ± | 0.0038 | 0.0021 | ± | 0.0042 |
| **Kidneys** | 0.0050 | ± | 0.0049 | 0.0010 | ± | 0.0002 | 0.0032 | ± | 0.0016 | 0.0026 | ± | 0.0021 |
| **Ovaries** | 0.0052 | ± | 0.0054 | 0.0012 | ± | 0.0009 | 0.0031 | ± | 0.0028 | 0.0040 | ± | 0.0042 |
| **Bone (femur)** | 0.0072 | ± | 0.0053 | 0.0165 | ± | 0.0153 | 0.0283 | ± | 0.0386 | 0.0065 | ± | 0.0089 |
| **Muscle** | 0.0084 | ± | 0.0092 | 0.0022 | ± | 0.0032 | 0.0030 | ± | 0.0049 | 0.0036 | ± | 0.0056 |
| **Stomach (empty)** | 3.83 | ± | 4.36 | 0.95 | ± | 1.14 | 0.16 | ± | 0.17 | 0.07 | ± | 0.04 |
| **Stomach contents** | 54.47 | ± | 34.37 | 11.78 | ± | 12.43 | 0.86 | ± | 0.86 | 1.12 | ± | 0.96 |
| **S. intestine (empty)** | 0.69 | ± | 0.35 | 0.30 | ± | 0.31 | 0.02 | ± | 0.01 | 0.02 | ± | 0.02 |
| **S. intestine contents** | 23.38 | ± | 11.94 | 10.18 | ± | 13.25 | 0.58 | ± | 0.36 | 0.67 | ± | 0.76 |
| **L. intestine (empty)** | 1.07 | ± | 0.65 | 0.84 | ± | 0.45 | 0.03 | ± | 0.03 | 0.07 | ± | 0.06 |
| **L. intestine contents** | 21.56 | ± | 7.83 | 22.04 | ± | 10.70 | 1.73 | ± | 2.73 | 1.59 | ± | 1.51 |

**Table S11.** Biodistribution of 1 µm [$^{89}$Zr]Zr-DFO-PS particles (1.85 MBq. 0.1 mg. in 0.1 ml of PBS) after oral administration via gavage to healthy, immunocompetent mice. Values are in units of %ID and are expressed as mean ± standard deviation.

| | **%ID (percentage of injected dose)** | | | | | | | | | | | |
|---|---|---|---|---|---|---|---|---|---|---|---|---|
| **Organ** | 6 h | | | 12 h | | | 24 h | | | 48 h | | |
| **Urine** | 0.00080 | ± | 0.00056 | 0.00046 | ± | 0.00038 | 0.00075 | ± | 0.00078 | 0.00075 | ± | 0.00078 |
| **Blood** | 0.00024 | ± | 0.00023 | 0.00034 | ± | 0.00035 | 0.00022 | ± | 0.00039 | 0.00040 | ± | 0.00057 |
| **Gallbladder** | 0.00020 | ± | 0.00010 | 0.00020 | ± | 0.00012 | 0.00043 | ± | 0.00059 | 0.00060 | ± | 0.00085 |
| **Heart** | 0.00006 | ± | 0.00009 | 0.00016 | ± | 0.00005 | 0.00032 | ± | 0.00066 | 0.00042 | ± | 0.00056 |
| **Lungs** | 0.00018 | ± | 0.00013 | 0.00016 | ± | 0.00005 | 0.00030 | ± | 0.00057 | 0.00032 | ± | 0.00045 |
| **Liver** | 0.00324 | ± | 0.00306 | 0.00032 | ± | 0.00018 | 0.00046 | ± | 0.00053 | 0.00038 | ± | 0.00064 |
| **Spleen** | 0.00020 | ± | 0.00012 | 0.00014 | ± | 0.00009 | 0.00046 | ± | 0.00076 | 0.00040 | ± | 0.00063 |
| **Pancreas** | 0.00026 | ± | 0.00037 | 0.00008 | ± | 0.00011 | 0.00028 | ± | 0.00041 | 0.00022 | ± | 0.00044 |
| **Kidneys** | 0.00146 | ± | 0.00141 | 0.00032 | ± | 0.00004 | 0.00096 | ± | 0.00052 | 0.00066 | ± | 0.00055 |
| **Ovaries** | 0.00096 | ± | 0.00119 | 0.00030 | ± | 0.00021 | 0.00054 | ± | 0.00048 | 0.00060 | ± | 0.00064 |
| **Bone (femur)** | 0.00012 | ± | 0.00008 | 0.00020 | ± | 0.00019 | 0.00038 | ± | 0.00054 | 0.00036 | ± | 0.00070 |
| **Muscle** | 0.00062 | ± | 0.00076 | 0.00012 | ± | 0.00011 | 0.00030 | ± | 0.00046 | 0.00026 | ± | 0.00042 |
| **Stomach (empty)** | 0.498 | ± | 0.595 | 0.125 | ± | 0.143 | 0.035 | ± | 0.053 | 0.009 | ± | 0.006 |
| **Stomach contents** | 40.331 | ± | 18.882 | 6.233 | ± | 6.119 | 0.199 | ± | 0.213 | 0.164 | ± | 0.127 |
| **S. intestine (empty)** | 0.358 | ± | 0.196 | 0.193 | ± | 0.218 | 0.008 | ± | 0.004 | 0.009 | ± | 0.008 |
| **S. intestine contents** | 12.271 | ± | 5.713 | 6.479 | ± | 8.966 | 0.331 | ± | 0.191 | 0.216 | ± | 0.243 |
| **L. intestine (empty)** | 0.259 | ± | 0.144 | 0.235 | ± | 0.123 | 0.008 | ± | 0.008 | 0.016 | ± | 0.015 |
| **L. intestine contents** | 8.629 | ± | 4.000 | 10.937 | ± | 5.045 | 0.943 | ± | 1.500 | 0.881 | ± | 0.944 |

**Table S12.** Biodistribution of 6 μm [[89]Zr]Zr-DFO-PS particles (1.85 MBq. 0.1 mg. in 0.1 ml of PBS) after oral administration via gavage to healthy, immunocompetent mice. Values are in units of %ID/g and are expressed as mean ± standard deviation.

| **%ID/g (percentage of injected dose per gram of organ)** | | | | | | | | | | | | |
|---|---|---|---|---|---|---|---|---|---|---|---|---|
| **Organ** | | 6 h | | | 12 h | | | 24 h | | | 48 h | |
| **Urine** | 0.0098 | ± | 0.0050 | 0.0112 | ± | 0.0045 | 0.1066 | ± | 0.1995 | 0.0218 | ± | 0.0184 |
| **Blood** | 0.0005 | ± | 0.0003 | 0.0006 | ± | 0.0005 | 0.0017 | ± | 0.0016 | 0.0025 | ± | 0.0028 |
| **Gallbladder** | 0.3143 | ± | 0.4859 | 0.0557 | ± | 0.0508 | 0.2109 | ± | 0.2270 | 0.6522 | ± | 0.5239 |
| **Heart** | 0.0017 | ± | 0.0007 | 0.0006 | ± | 0.0009 | 0.0050 | ± | 0.0039 | 0.0097 | ± | 0.0120 |
| **Lungs** | 0.0009 | ± | 0.0009 | 0.0007 | ± | 0.0006 | 0.0041 | ± | 0.0031 | 0.0057 | ± | 0.0061 |
| **Liver** | 0.0004 | ± | 0.0004 | 0.0015 | ± | 0.0029 | 0.0006 | ± | 0.0006 | 0.0009 | ± | 0.0008 |
| **Spleen** | 0.0028 | ± | 0.0051 | 0.0023 | ± | 0.0041 | 0.0106 | ± | 0.0094 | 0.0335 | ± | 0.0479 |
| **Pancreas** | 0.0016 | ± | 0.0011 | 0.0006 | ± | 0.0007 | 0.0033 | ± | 0.0031 | 0.0082 | ± | 0.0077 |
| **Kidneys** | 0.0007 | ± | 0.0003 | 0.0103 | ± | 0.0196 | 0.0030 | ± | 0.0021 | 0.0059 | ± | 0.0044 |
| **Ovaries** | 0.0009 | ± | 0.0006 | 0.0040 | ± | 0.0071 | 0.0049 | ± | 0.0027 | 0.0102 | ± | 0.0074 |
| **Bone (femur)** | 0.0061 | ± | 0.0047 | 0.0084 | ± | 0.0090 | 0.0640 | ± | 0.0212 | 0.0205 | ± | 0.0091 |
| **Muscle** | 0.0027 | ± | 0.0030 | 0.0028 | ± | 0.0022 | 0.0057 | ± | 0.0067 | 0.0141 | ± | 0.0127 |
| **Stomach (empty)** | 4.883 | ± | 6.680 | 0.331 | ± | 0.380 | 0.016 | ± | 0.011 | 0.066 | ± | 0.026 |
| **Stomach contents** | 62.494 | ± | 26.189 | 12.748 | ± | 12.732 | 0.119 | ± | 0.086 | 1.620 | ± | 0.771 |
| **S. intestine (empty)** | 0.344 | ± | 0.197 | 0.307 | ± | 0.400 | 0.003 | ± | 0.001 | 0.014 | ± | 0.007 |
| **S. intestine contents** | 8.163 | ± | 3.201 | 9.872 | ± | 8.788 | 0.031 | ± | 0.012 | 0.551 | ± | 0.135 |
| **L. intestine (empty)** | 0.316 | ± | 0.168 | 0.691 | ± | 0.255 | 0.011 | ± | 0.006 | 0.049 | ± | 0.019 |
| **L. intestine contents** | 14.703 | ± | 5.733 | 21.997 | ± | 10.086 | 0.105 | ± | 0.063 | 0.616 | ± | 0.209 |

**Table S13.** Biodistribution of 6 µm [$^{89}$Zr]Zr-DFO-PS particles (1.85 MBq. 0.1 mg. in 0.1 ml of PBS) after oral administration via gavage to healthy, immunocompetent mice. Values are in units of %ID and are expressed as mean ± standard deviation.

| | %ID (percentage of injected dose) | | | |
|---|---|---|---|---|
| **Organ** | 6 h | 12 h | 24 h | 48 h |
| **Urine** | 0.00073 ± 0.00040 | 0.00028 ± 0.00024 | 0.00063 ± 0.00036 | 0.00135 ± 0.00099 |
| **Blood** | 0.00014 ± 0.00009 | 0.00012 ± 0.00013 | 0.00054 ± 0.00057 | 0.00096 ± 0.00105 |
| **Gallbladder** | 0.00020 ± 0.00000 | 0.00010 ± 0.00000 | 0.00070 ± 0.00017 | 0.00115 ± 0.00123 |
| **Heart** | 0.00016 ± 0.00005 | 0.00006 ± 0.00009 | 0.00044 ± 0.00035 | 0.00086 ± 0.00109 |
| **Lungs** | 0.00014 ± 0.00017 | 0.00012 ± 0.00011 | 0.00068 ± 0.00047 | 0.00094 ± 0.00104 |
| **Liver** | 0.00040 ± 0.00036 | 0.00180 ± 0.00347 | 0.00072 ± 0.00066 | 0.00100 ± 0.00095 |
| **Spleen** | 0.00018 ± 0.00035 | 0.00016 ± 0.00025 | 0.00060 ± 0.00052 | 0.00090 ± 0.00115 |
| **Pancreas** | 0.00016 ± 0.00011 | 0.00006 ± 0.00009 | 0.00036 ± 0.00036 | 0.00080 ± 0.00087 |
| **Kidneys** | 0.00018 ± 0.00011 | 0.00282 ± 0.00537 | 0.00082 ± 0.00049 | 0.00154 ± 0.00115 |
| **Ovaries** | 0.00016 ± 0.00011 | 0.00036 ± 0.00053 | 0.00080 ± 0.00031 | 0.00150 ± 0.00106 |
| **Bone (femur)** | 0.00010 ± 0.00007 | 0.00012 ± 0.00011 | 0.00084 ± 0.00030 | 0.00124 ± 0.00099 |
| **Muscle** | 0.00016 ± 0.00015 | 0.00020 ± 0.00019 | 0.00058 ± 0.00070 | 0.00106 ± 0.00098 |
| **Stomach (empty)** | 0.610 ± 0.783 | 0.047 ± 0.053 | 0.002 ± 0.001 | 0.007 ± 0.003 |
| **Stomach contents** | 39.131 ± 9.687 | 7.893 ± 8.754 | 0.013 ± 0.009 | 0.103 ± 0.054 |
| **S. intestine (empty)** | 0.153 ± 0.093 | 0.171 ± 0.240 | 0.002 ± 0.000 | 0.006 ± 0.003 |
| **S. intestine contents** | 4.597 ± 1.691 | 6.757 ± 6.116 | 0.012 ± 0.004 | 0.223 ± 0.058 |
| **L. intestine (empty)** | 0.065 ± 0.032 | 0.193 ± 0.067 | 0.003 ± 0.001 | 0.011 ± 0.004 |
| **L. intestine contents** | 5.615 ± 1.276 | 10.826 ± 9.211 | 0.059 ± 0.034 | 0.299 ± 0.095 |
